# A Quantitative Analysis of Cellular Lipid Compositions During Acute Proteotoxic ER Stress Reveals Specificity in the Production of Asymmetric Lipids

**DOI:** 10.3389/fcell.2020.00756

**Published:** 2020-08-04

**Authors:** John Reinhard, Carsten Mattes, Kristina Väth, Toni Radanović, Michal A. Surma, Christian Klose, Robert Ernst

**Affiliations:** ^1^Medical Biochemistry and Molecular Biology, Medical Faculty, Saarland University, Homburg, Germany; ^2^PZMS, Center for Molecular Signaling, Medical Faculty, Saarland University, Homburg, Germany; ^3^Lipotype GmbH, Dresden, Germany

**Keywords:** UPR, Ire1, lipid bilayer stress, proteotoxic stress, lipidomics, DTT, tunicamycin, asymmetric lipids

## Abstract

The unfolded protein response (UPR) is central to endoplasmic reticulum (ER) homeostasis by controlling its size and protein folding capacity. When activated by unfolded proteins in the ER-lumen or aberrant lipid compositions, the UPR adjusts the expression of hundreds of target genes to counteract ER stress. The proteotoxic drugs dithiothreitol (DTT) and tunicamycin (TM) are commonly used to induce misfolding of proteins in the ER and to study the UPR. However, their potential impact on the cellular lipid composition has never been systematically addressed. Here, we report the quantitative, cellular lipid composition of *Saccharomyces cerevisiae* during acute, proteotoxic stress in both rich and synthetic media. We show that DTT causes rapid remodeling of the lipidome when used in rich medium at growth-inhibitory concentrations, while TM has only a marginal impact on the lipidome under our conditions of cultivation. We formulate recommendations on how to study UPR activation by proteotoxic stress without interferences from a perturbed lipid metabolism. Furthermore, our data suggest an intricate connection between the cellular growth rate, the abundance of the ER, and the metabolism of fatty acids. We show that *Saccharomyces cerevisiae* can produce asymmetric lipids with two saturated fatty acyl chains differing substantially in length. These observations indicate that the pairing of saturated fatty acyl chains is tightly controlled and suggest an evolutionary conservation of asymmetric lipids and their biosynthetic machineries.

## Introduction

Biological membranes are complex assemblies of proteins and lipids forming the boundary of cellular life and compartmentalizing biochemical processes in different organelles (van Meer et al., 2008; Bigay and Antonny, 2012). A major fraction of cellular bioactivity is localized to biological membranes: one third of all proteins and the majority of therapeutic drug targets are either membrane embedded or membrane associated (Uhlén et al., 2015). The interactions, activities, and subcellular localizations of membrane proteins are modulated by their complex and dynamically regulated environment (Lee, 2004; Phillips et al., 2009; Lorent et al., 2017). The lipidome of a eukaryotic cell comprises hundreds, if not thousands, of lipid species and it can be remodeled upon dietary perturbation, by the growth phase, and in response to external cues such as temperature or nutrient availability (Shevchenko and Simons, 2010; Klose et al., 2012; Casanovas et al., 2015; Han, 2016; Levental et al., 2020). This membrane responsiveness down to the level of individual lipid species is essential to sustain cellular fitness, by maintaining physicochemical membrane properties such as fluidity, permeability, phase behavior, and surface charge density in a regime acceptable for membrane function (Bigay and Antonny, 2012; Sezgin et al., 2017; Ernst et al., 2018; Harayama and Riezman, 2018). Our understanding of these complex remodeling processes, their purposes and the underlying principles, remains rather limited.

The endoplasmic reticulum (ER) is the central hub for membrane biogenesis in eukaryotic cells (van Meer et al., 2008). The vast majority of membrane proteins is targeted to ER-localized machineries for membrane insertion (Aviram and Schuldiner, 2017). Likewise, a major fraction of membrane lipids including sterols, glycerophospholipids, and ceramides is produced in the ER (van Meer et al., 2008). In the past years it became increasingly clear that protein quality control and lipid metabolism are intimately connected both on the cellular and the molecular level (Jonikas et al., 2009; De Kroon et al., 2013; Stordeur et al., 2014; Volmer and Ron, 2015; Fun and Thibault, 2020; Goder et al., 2020).

A prominent example is the unfolded protein response (UPR) (Walter and Ron, 2011). Both an accumulation of unfolded protein in the lumen of the ER and stiffening of the ER membrane due to lipid imbalances serve as activating signals for the UPR (Halbleib et al., 2017; Karagöz et al., 2017; Adams et al., 2019; Preissler and Ron, 2019). How precisely these activating signals from the lumen of the ER and the ER membrane are integrated by the transducers of the UPR is matter of active debate (Volmer and Ron, 2015; Covino et al., 2018; Fun and Thibault, 2020). Once activated, the UPR increases the size of the ER and its folding capacity in order to reestablish ER homeostasis even under adverse conditions (Bernales et al., 2006; Schuck et al., 2009). This is accomplished by a global attenuation of protein production (Walter and Ron, 2011), by upregulating the ER-associated degradation machinery, and by increasing the number of ER chaperones (Cox et al., 1993; Jonikas et al., 2009). At the same time, the UPR induces the expression of a large number of genes involved in membrane-related processes such as lipid biosynthesis, membrane protein sorting, and vesicular traffic (Travers et al., 2000). Unbiased genetic screens and targeted perturbations of lipid metabolism have clearly established the mutual dependency of the UPR and lipid metabolism (Jonikas et al., 2009; Pineau et al., 2009; Schuck et al., 2009; Promlek et al., 2011; Thibault et al., 2012; Surma et al., 2013). Given its central importance for ER homeostasis and cell physiology, it is not surprising that the UPR plays also a crucial role in pathologic processes such as viral infections, neurodegeneration, and cancer (Wang and Kaufman, 2012,Wang and Kaufman, 2014; Hetz et al., 2019). Metabolic diseases associated with chronic UPR signaling such as type II diabetes and non-alcoholic steatohepatitis (Kaufman, 2002; Fonseca et al., 2009) are historically studied with a focus on the role of unfolded, soluble proteins in the ER lumen while the contribution of signals from the ER membrane remains understudied.

The eukaryotic model organism *Saccharomyces cerevisiae* (*S. cerevisiae*) has facilitated the identification of numerous key components of the secretory pathway, lipid metabolism, and the proteostasis network (Novick et al., 1980; Wolf and Schäfer, 2006; Henry et al., 2012; De Kroon et al., 2013). In contrast to metazoans, the UPR in *S. cerevisiae* relies on a single, ER-localized UPR transducer (Kimata and Kohno, 2011): the Inositol-requiring enzyme 1 (Ire1p). It is conserved from yeast to humans and comprises an N-terminal sensor domain facing the ER-lumen, a single transmembrane helix, and cytosolic effector domains with kinase and RNase functions. The formation of dimers and higher oligomers of Ire1p during stress from unfolded proteins or from the ER membrane (Kimata et al., 2007; Korennykh et al., 2009; Halbleib et al., 2017) triggers the *trans*-autophosphorylation of the cytosolic kinase domain and the activation of the adjacent RNase domain. The RNase activity of Ire1p contributes to an unconventional splicing of the *HAC1* mRNA in the cytosol (Cox and Walter, 1996; Mori et al., 1996) thereby facilitating the production of the active transcription factor Hac1p regulating several hundred UPR-target genes.

For studying and assaying the UPR, it is common practice to stress the cells acutely either with dithiothreitol (DTT), a reducing agent interfering with disulfide bridge formation in the ER-lumen, or tunicamycin (TM), a natural inhibitor of the N-linked glycosylation of proteins in the ER (Azim and Surani, 1979). It is generally assumed that DTT and TM exclusively cause proteotoxic ER stress. However, the impact of DTT and TM on the cellular lipid composition has never been systematically tested.

Here, we have studied the impact of DTT or TM on the lipidome of *S. cerevisiae* in both rich and synthetic medium. Serendipitously, we find evidence for a remarkable selectivity of *S. cerevisiae* in the generation and metabolism of highly asymmetric glycerophospholipids with one saturated, medium fatty acyl chain (C10 or C12) and a long, saturated one (C16 or C18). Despite a high overall abundance of unsaturated fatty acyl chains (C16:1 or C18:1), we find an almost exclusive paring of two saturated fatty acyl chains in these asymmetric glycerophospholipids, thereby implying a strong, inherent selectivity of acyl chain pairing. With respect to the UPR, we find that (1) DTT and TM impair cellular growth, (2) the medium has a significant impact on the cellular lipidome thereby potentially tuning the sensitivity of the UPR, (3) DTT at growth-inhibitory concentrations causes a substantial and rapid remodeling of the lipidome in rich medium, and (4) TM under our experimental conditions has only a marginal impact on the cellular lipidome in both synthetic and rich medium. Based on these findings, we provide a guideline to predictably and unambiguously activate the UPR by proteotoxic stress, whilst minimizing potential artifacts from lipid bilayer stress.

## Materials and Methods

### Yeast Strains

Yeasts used were the standard laboratory wild-type *S. cerevisiae* strain BY4741 MATa *his3*Δ1 *leu2*Δ0 *met15*Δ0 *ura3*Δ0 and the *ire1*Δ-derivative BY4741 MATa; *ura3*Δ0; *leu2*Δ0; *his3*Δ1; *met15*Δ0; *YHR079c*::*kan*MX4.

### Reagents

Chemicals and solvents of HPLC/LC-MS analytical grade were used. TM (#T7765), DTT (#D0632), and ammonium bicarbonate (#9830) were purchased from Sigma-Aldrich. Ammonium sulfate (#9218) was purchased from Carl Roth.

### Media

All media were prepared according to standard protocols (Dymond, 2013). D(+)-Glucose (#HN06, tryptone/peptone (#8952), and yeast extract (#2363) were purchased from Carl Roth. Yeast nitrogen base (YNB) (#CYN0602), agar–agar (#AGA03), and complete supplement mixture (CSM complete) (#DCS0019) were purchased from FORMEDIUM.

### Cell Cultivation for Lipidomics

Cells were cultivated under constant agitation at 30°C at 220 rpm, if not stated otherwise. Every lipidomic sample started from an individual, single colony on either yeast peptone dextrose (YPD) or synthetic complete dextrose (SCD) agar plates. A single colony was used to inoculate a pre-culture, which was then cultivated overnight for 21 h in either YPD or SCD liquid medium. The resulting stationary culture was used to inoculate a main culture in fresh medium to a final OD_600_ of 0.1. When the culture reached an OD_600_ of 0.8 ± 0.05, the cells were either stressed with DTT, TM or left untreated. DTT was used at a final concentration 8 mM and 2 mM in YPD and SCD, respectively. TM was used at a final concentration of 1.0 μg/ml and 1.5 μg/ml in YPD and SCD, respectively. After an additional hour of cultivation, 20 OD units of cells were harvested by centrifugation (3,500 × *g*, 5 min, 4°C), and washed three-times with ice-cold 155 mM ammonium bicarbonate supplemented with 10 mM sodium azide in 1.5 ml reaction tubes using quick centrifugation (10.000 × *g*, 20 s, 4°C). The resulting cell pellets were snap-frozen with liquid nitrogen and stored for up to 4 weeks at −80°C. Prior to cell lysis, pellets were thawed on ice and then resuspended in 1 ml 155 mM ammonium bicarbonate. 200 μl zirconia beads were added to the suspension and cells were disrupted by vigorous shaking using a DisruptorGenie for 10 min at 4°C. 500 μl of the resulting lysate was snap-frozen and used for further analysis via shotgun mass spectrometry.

### Growth Assay – Acute Stress

Cultures in YPD and SCD were inoculated precisely as described for lipidomic experiments. The density of the culture was monitored over a prolonged period of time by determining the OD_600_ for up to 5 h after they had reached an OD_600_ = 0.8. For determining the doubling time of an exponentially growing culture, all data points with an OD_600_ between 0.2 and 2.5 were considered. The data were fitted to the exponential (Malthusian) growth function using Prism 8 for macOS Version 8.4.1.

### Growth Assay – Prolonged Stress

Stationary overnight cultures in YPD were used to inoculate a pre-culture in either YPD or SCD to an OD_600_ of 0.2. The cells were then cultivated for 6 h to reach the exponential growth phase. These cultures were used to inoculate a main culture in a 96-well plate to an OD_600_ of 0.01 using fresh medium (either YPD or SCD) containing different concentrations of DTT. After cultivation for 16 h at 30°C with no agitation, the final OD_600_ was determined after intense mixing of the culture using a microplate reader (Tecan Microplate Reader Spark).

### Lipid Extraction for Mass Spectrometry Lipidomics

Mass spectrometry-based lipid analysis was performed by Lipotype GmbH (Dresden, Germany) as described (Ejsing et al., 2009; Klose et al., 2012). Lipids were extracted using a two-step chloroform/methanol procedure (Ejsing et al., 2009). Samples were spiked with internal lipid standard mixture containing: CDP-DAG 17:0/18:1, ceramide 18:1;2/17:0 (Cer), diacylglycerol 17:0/17:0 (DAG), lyso-phosphatidate 17:0 (LPA), lyso-phosphatidylcholine 12:0 (LPC), lyso-phosphatidylethanolamine 17:1 (LPE), lyso-phosphatidylinositol 17:1 (LPI), lyso-phosphatidylserine 17:1 (LPS), phosphatidate 17:0/14:1 (PA), phosphatidylcholine 17:0/14:1 (PC), phosphatidylethanolamine 17:0/14:1 (PE), phosphatidylglycerol 17:0/14:1 (PG), phosphatidylinositol 17:0/14:1 (PI), phosphatidylserine 17:0/14:1 (PS), ergosterol ester 13:0 (EE), triacylglycerol 17:0/17:0/17:0 (TAG), stigmastatrienol, inositolphosphorylceramide 44:0;2 (IPC), mannosyl-inositolphosphorylceramide 44:0;2 (MIPC), mannosyl-di-(inositolphosphoryl)ceramide 44:0;2 (M(IP)_2_C). After extraction, the organic phase was transferred to an infusion plate and dried in a speed vacuum concentrator. 1st step dry extract was resuspended in 7.5 mM ammonium acetate in chloroform/methanol/propanol (1:2:4, V:V:V) and 2nd step dry extract in 33% ethanol solution of methylamine in chloroform/methanol (0.003:5:1; V:V:V). All liquid handling steps were performed using Hamilton Robotics STARlet robotic platform with the Anti Droplet Control feature for organic solvents pipetting.

### MS Data Acquisition

Samples were analyzed by direct infusion on a QExactive mass spectrometer (Thermo Scientific) equipped with a TriVersa NanoMate ion source (Advion Biosciences). Samples were analyzed in both positive and negative ion modes with a resolution of *R*_m/z = 200_ = 280000 for MS and *R*_m/z = 200_ = 17500 for MSMS experiments, in a single acquisition. MSMS was triggered by an inclusion list encompassing corresponding MS mass ranges scanned in 1 Da increments (Surma et al., 2015). Both MS and MSMS data were combined to monitor EE, DAG, and TAG ions as ammonium adducts; PC as an acetate adduct; and CL, PA, PE, PG, PI, and PS as deprotonated anions. MS only was used to monitor LPA, LPE, LPI, LPS, IPC, MIPC, M(IP)_2_C as deprotonated anions; Cer and LPC as acetate adducts and ergosterol as protonated ion of an acetylated derivative (Liebisch et al., 2006).

### MS Data Analysis

Data were analyzed by Lipotype GmbH using an in-house developed lipid identification software based on LipidXplorer (Herzog et al., 2011, 2012). Data post-processing and normalization were performed using an in-house developed data management system. Only lipid identifications with a signal-to-noise ratio > 5, and a signal intensity five-fold higher than in corresponding blank samples were considered for further data analysis.

## Results

We investigated the impact of two potent, proteotoxic inducers of ER stress, namely DTT and TM, on cellular growth and the cellular lipid composition. Our ultimate goal was to faithfully induce proteotoxic stress in the lumen of the ER whilst minimizing potential artifacts from aberrant ER lipid compositions.

### DTT and TM Inhibit Cellular Growth

**FIGURE 1:**
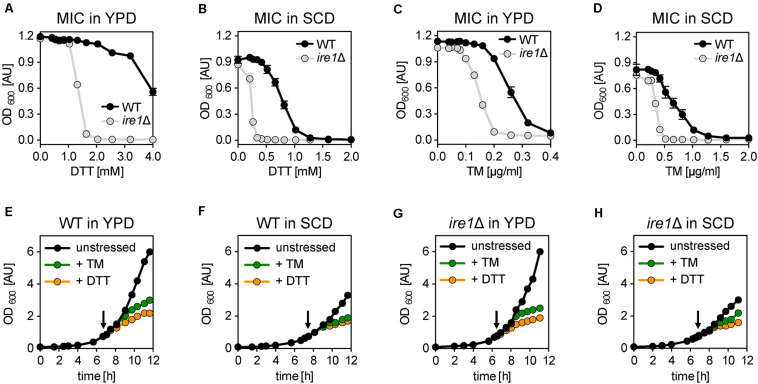
Acute and prolonged ER stress inhibits cellular growth. **(A–D)** Determination of the minimal inhibitory concentration (MIC) of DTT and TM for the indicated strains. An overnight culture of the indicated strains in rich medium (YPD) was used to inoculate a main culture in either YPD **(E)** or SCD **(F)** to an OD_600_ of 0.2. After 6 h of cultivation to reach the exponential growth phase, a fresh culture in a 96-well plate was inoculated to an OD_600_ = 0.01 and adjusted to the indicated, final concentrations of either DTT or TM. After 16 h of cultivation, the OD_600_ as a measure for the overnight growth was plotted against the concentration of the proteotoxic drug. The data in panels **(A–D)** are plotted as the average ± standard deviation (SD) from three independent experiments (*n* = 3) with technical duplicates. **(E–H)** BY4741 WT and the isogenic *ire1*Δ strain were cultivated in either synthetic (SCD) or rich (YPD) medium at 30°C. The main cultures were inoculated to an OD_600_ of 0.1 from a stationary overnight culture in the respective medium. Cell proliferation is monitored by plotting the OD_600_ against the time of cultivation. At an OD_600_ of 0.80 ± 0.05 the cells were either left untreated (black) or stressed with either TM (green) or DTT (orange). An arrow indicates the time point of drug addition. DTT was used at a final concentration 8 mM and 2 mM in YPD and SCD, respectively. TM was used at a final concentration of 1.0 μg/ml and 1.5 μg/ml in YPD and SCD, respectively. The data in panels **(A–D)** are from a single, representative experiment. Raw data can be found in the Supplementary Data Sheet 2.

We wanted to know the impact of DTT and TM on cellular growth. To this end, we cultivated the *S. cerevisiae* wildtype (WT) strain BY4741 and isogenic *ire1*Δ cells in either rich medium (YPD) or synthetic medium (SCD) to the exponential growth phase. Using these cells, we inoculated fresh cultures in a 96-well plate to an OD_600_ of 0.01 in the respective medium supplemented with various concentrations of DTT and TM. After overnight cultivation, cellular growth was assayed via the OD_600_ (Figures 1A–D). Not surprisingly, WT cells are more resistant to DTT- or TM-induced ER stress than *ire1*Δ in both rich and synthetic medium (Figures 1A–D). This suggests that UPR-activation via Ire1p contributes to cellular fitness under conditions of prolonged proteotoxic stress. Notably, the choice of the medium affects the growth-inhibitory concentrations of DTT and TM such that higher initial concentrations of DTT are required to inhibit overnight growth in rich medium, while lower concentrations are sufficient in minimal medium (Figures 1A,B). In contrast, lower concentrations of TM are required to block overnight growth in rich versus synthetic medium (Figures 1C,D). The underlying reasons remain unclear. Among other possibilities, the medium might affect the drug *per se* (e.g., DTT oxidation), the uptake and extrusion of the compound, or -via diverse mechanisms- the cellular resistance to proteotoxic stress. Nevertheless, our data help choosing appropriate concentrations to effectively inhibit overnight growth for each medium and both drugs.

Next, we wanted to study the impact of acute ER stress on cellular growth. We cultivated WT cells in rich (YPD) and synthetic (SCD) medium (Figures 1E,F) under conditions most commonly used for studying the biology of *S. cerevisiae* (Sherman, 2002). We inoculated liquid cultures to an OD_600_ of 0.1 using stationary, overnight cultures in the respective medium and then followed the cellular growth over time. When the cultures reached an OD_600_ of 0.75 to 0.8, the cells were either left untreated or stressed with DTT or TM at concentrations causing a near-complete inhibition of overnight growth to account for the different dose–response curves in different media (Figures 1A–D). Specifically, DTT was used at a concentration of 8 mM and 2 mM, while TM was used at a concentration of 1.0 μg/ml and 1.5 μg/ml in rich and synthetic medium, respectively. Notably, 8 mM of DTT has previously been used to study ER membrane expansion in stressed cells, while 1–2 μg/ml of TM are known to reorganize Golgi traffic and mitochondrial enlargement by activating the UPR (Bernales et al., 2007; Schuck et al., 2009; Hsu et al., 2016; Tran et al., 2019).

Expectedly, we find that unstressed cells grow faster in rich medium (doubling time 86 min) than in synthetic medium (doubling time 107 min) (Figures 1E,F), which underscores the previous finding that BY4741 strains requires an additional supplementation of the SCD medium for optimal growth (Hanscho et al., 2012). Furthermore, DTT- and TM-stressed cells grow markedly slower compared to the unstressed cells in both media (Figures 1E,F). Consistent with previous observations (Pincus et al., 2010), the reduced rate of growth becomes apparent as early as 1 h after the addition of the stress-inducing agents (Supplementary Figures S1A,B). Notably, the impact of DTT is more pronounced than the impact of TM at the given concentrations (Figures 1E,F).

Next, we wanted to test if the reduced growth of the stressed cells is due to an activation of the UPR, which is known to peak within 1 h after the addition of DTT or TM to the medium and which largely remodels the cellular transcriptome (Kawahara et al., 1997; Travers et al., 2000; Promlek et al., 2011). Surprisingly, the growth of both stressed and unstressed *ire1*Δ cells was indistinguishable from WT cells in both rich and synthetic medium (Figures 1E–H and Supplementary Figures S1C–H). This suggests that DTT and TM impair cellular growth during this early phase of stress predominantly via their impact on protein folding and not by processes downstream of UPR activation. The slightly higher potency of DTT to impede cellular growth compared to TM at the given concentrations may reflect the fact that these compounds affect the folding of different sets of proteins: proteins with disulfide bonds in the case of DTT and N-linked glycosylated proteins in the case of TM. Furthermore, DTT can reduce already formed disulfide bonds and is known to directly affect also other cellular processes outside the ER such as the protein import into mitochondria (Mesecke et al., 2005) and protein palmitoylation (Levental et al., 2010). In contrast, TM affects only the glycosylation of freshly synthesized proteins. Our data suggest that the growth inhibition observed in *acutely* stressed cells is independent of UPR activation.

### Experimental Outline and Global Insights From Principal Component Analysis

**FIGURE 2:**
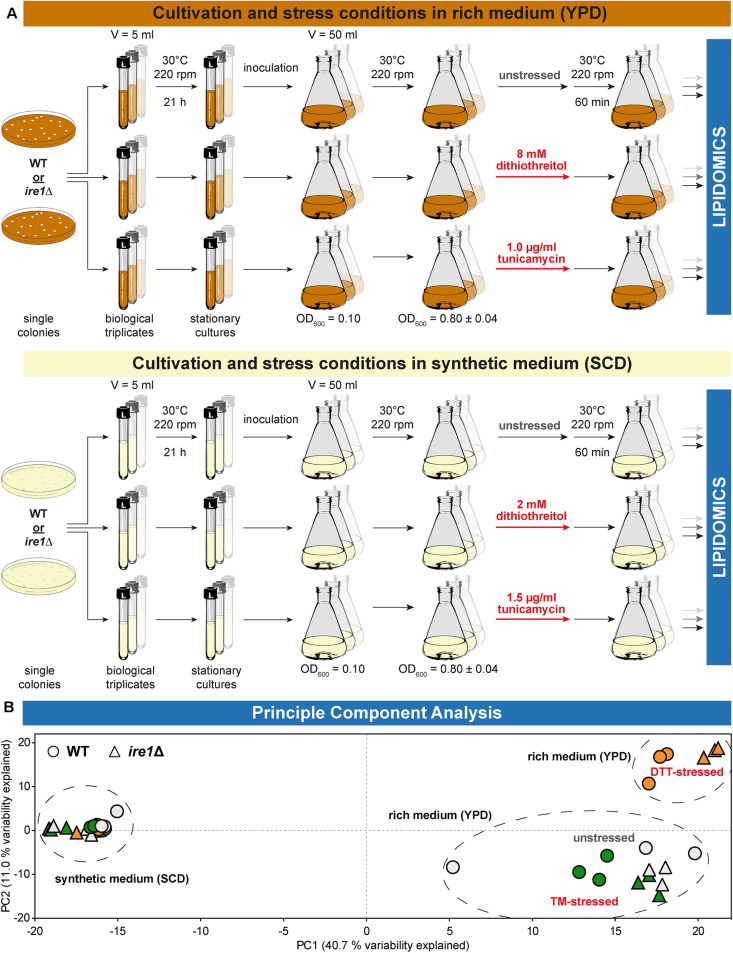
Experimental conditions affecting lipidome variability. **(A)** Overview of the cultivation and stress conditions use for lipidomic analysis. **(B)** A two-dimensional principal component analysis (PCA) of lipid species abundances reveals the degree of variations between different cultivation and stress conditions. Data from BY4741 WT and *ire1*Δ cells are indicated by circles and triangles, respectively. The color of the data points indicates unstressed cells in gray and cells stressed either with DTT (orange) or TM (green). Data from stressed and unstressed cells are indicated red and gray, respectively. PCA reveals that the lipidomes from both stressed and unstressed cells cultivated in synthetic medium (SCD) are very similar. In contrast, DTT induces a characteristic remodeling of the lipidome of both WT and *ire1*Δ cells cultivated in rich medium (YPD).

We used shotgun mass spectrometry-based lipidomics to comprehensively and quantitatively dissect the impact of acute proteotoxic stress on the cellular lipid composition. In light of the pronounced impact of DTT and TM on cellular growth, we focused on their immediate effects within 1 h of treatment. We analyzed the lipid composition for six conditions and two different strains each as biological triplicates (Figure 2A). A principal component analysis (PCA) of the entire dataset at the level of individual lipid species revealed a close clustering of all samples from WT and *ire1*Δ cells cultivated in SCD, thereby suggesting UPR activity itself has little impact on their lipidomes (Figure 2B). In contrast, we observed two clusters for cells cultivated in rich medium. One cluster contained samples from WT and *ire1*Δ cells that were either left untreated or stressed with TM, while the other cluster contained samples from cells that were stressed with DTT at concentrations commonly used for UPR activation. This suggests that DTT causes a substantial remodeling of the lipidome, while TM treatments have a lesser impact on the cellular lipid composition. Not surprisingly, the loadings plot suggests a correlation of specific groups of lipids (Supplementary Figure S2A). The total amount of lipids quantified from 1 OD unit of cells (Supplementary Figure S2B) and the amount of storage lipids (Supplementary Figure S2C) highlight a low variability between replicates and show that storage lipids are more abundant in synthetic (SCD) medium. Storages lipids comprise all triacylglycerol (TAG) species and ergosterol esters.

### The Impact of YPD and SCD Medium on the Lipidome of *S. cerevisiae*

**FIGURE 3:**
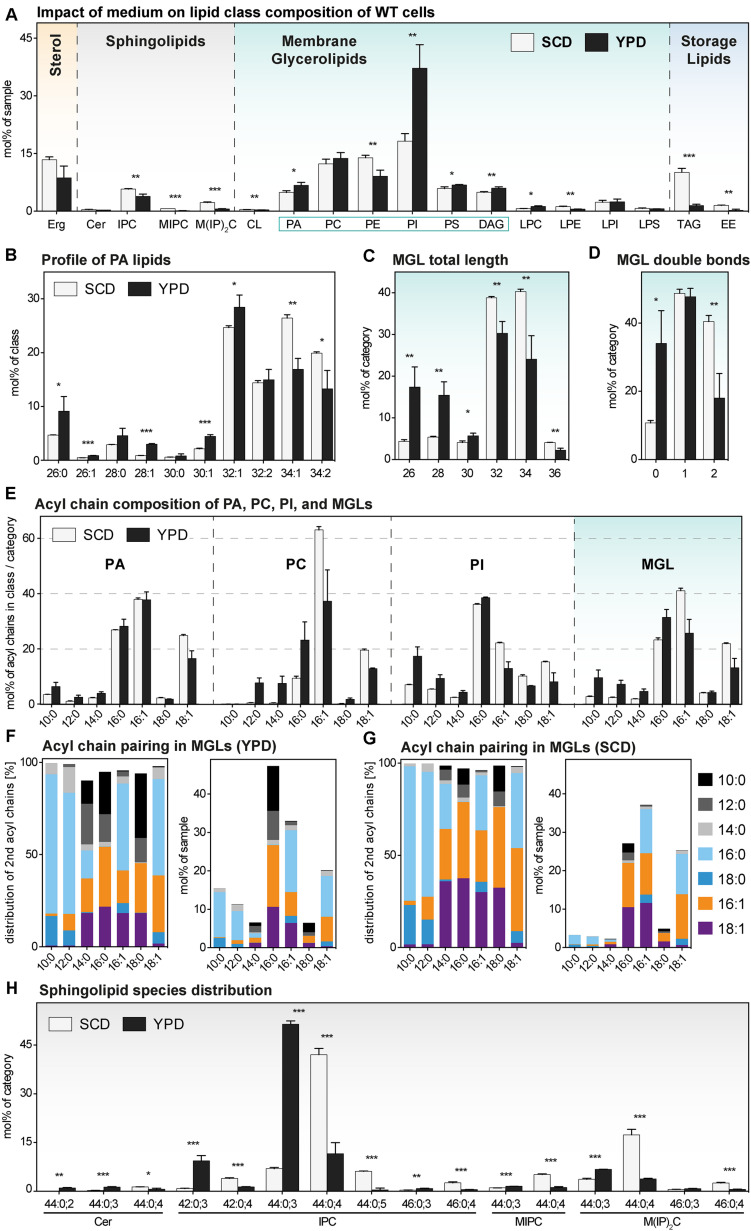
The lipid composition of *S. cerevisiae* WT indifferent media. A single colony of the BY4741 WT strain was used to inoculate a preculture in either synthetic (SCD) or rich (YPD) medium. After overnight cultivation for 21 h, a fresh culture was inoculated to an OD_600_ of 0.1. When the cells reached an OD_600_ of 0.80 ± 0.05, they were cultivated for one more hour. 20 OD equivalents of these cells were harvested and analyzed by lipid mass spectrometry. The data represented by black and white bars relate to cells cultivated in rich and synthetic medium, respectively [except for panels **(F** and **G)]**. **(A)** Lipid class composition in mol% of all quantified lipids in the sample organized by the categories: sterol (orange), sphingolipids (magenta), membrane glycerolipids (green, classes with two acyl chains highlighted by green box), and storage lipids (blue). Erg, ergosterol; Cer, ceramide; IPC, inositolphosphorylceramide; MIPC, mannosy-IPC; M(IP)2C, mannosyl-di-IPC; CL, cardiolipin; PA, phosphatidic acid; PC, phosphatidylcholine; PE, phosphatidylethanolamine; PI, phosphatidylinositol; PS, phosphatidylserine; DAG, diacylglycerol; LPC, lyso-PC; LPE, lyso-PE; LPI, lyso-PI; LPS, lyso-PS; TAG, triacylglycerol; EE, ergosteryl ester. **(B)** Profile of PA lipids in mol% of the class. **(C)** Total length of lipids in a sub-category of membrane glycerolipids (MGLs) including PA, PC, PE, PI, PS, DAG. The total length is given as the sum of carbon atoms in both fatty acyl chains in mol% of this sub-category. **(D)** Total number double bonds in a sub-category of MGLs (PA, PC, PE, PI, PS, DAG) is given as the sum of double bonds in both acyl chains, in mol% of this sub-category. **(E)** The acyl chain composition of PA, PC, PI and of a s sub-category of MGLs (PA, PC, PE, PI, PS, DAG) is normalized either to the individual lipid class or the sub-category and given in mol%. **(F,G)** The pairing of fatty acyl chains in MGLs (PA, PC, PE, PI, PS, DAG) is plotted for cells cultivated in **(F)** rich (YPD) and **(G)** synthetic (SCD) medium. The left panel indicates the pairing of fatty acyl chains in a sub-category of MGLs (PA, PC, PE, PI, PS, DAG) normalized to each particular fatty acyl chain and is given as mol%. The right panel indicates the abundance of acyl chain pairs in the sub-category of MGLs and is given in mol%. **(H)** Profile of sphingolipids in mol% of the category. The least abundant species in each panel are omitted for clarity. Each bar represents the average ± SD from *n* = 3 independent experiments. Statistical significance was tested by an unpaired two-tailed *t*-test using GraphPad Prism, *p < 0.05, **p < 0.01, ***p < 0.001.

For representing this complex dataset, we assorted the identified lipid species to one of four major lipid categories: sterols, sphingolipids (SLs), membrane glycerolipids (MGLs), and storage lipids. MGLs comprise all glycerophospholipids and diacylglycerol (DAG) species (Figure 3 and Supplementary Figure S3). Overall, we find a remarkable impact of the medium on the cellular lipid composition (Figure 3A).

#### Global Impact of the Medium on Sphingolipids

Yeast SLs comprise inositolphosphorylceramide (IPC), mannosyl-inositol phosphorylceramide (MIPC), mannosyl-di-(inositolphosphoryl) ceramide (M(IP)_2_C), and – less abundantly – ceramides (Cer). With the exception of Cer, all SLs have a significantly lower level in cells cultivated in rich medium compared to cells cultivated synthetic medium (Figure 3A). The same trend was observed for all SLs in *ire1*Δ cells (Supplementary Figure S3A). Thus, the lower level of sphingolipids in YPD-cultured cells is not due to possible differences in basal, constitutive UPR signaling. However, because SLs are concentrated along the secretory pathway and highly abundant in the plasma membrane (van Meer et al., 2008; Klemm et al., 2009; Hannich et al., 2011; Surma et al., 2011) it is tempting to speculate that cells cultivated in rich medium feature a higher abundance of inner membranes relative to the plasma membrane. In fact, the rapidly growing cells in rich medium have a particularly high demand for membrane biogenesis and both ER size and the rate of membrane lipid production can be controlled independently of UPR signaling (Loewen, 2004; Schuck et al., 2009; Henry et al., 2012).

#### Global Impact of the Medium on Membrane Glycerolipids

Membrane glycerolipids constitute the most abundant lipid category comprising the lipid classes phosphatidic acid (PA), phosphatidylcholine (PC), phosphatidyl-ethanolamine (PE), phosphatidylinositol (PI), phosphatidylserine (PS), and diacylglycerol (DAG) (Henry et al., 2012; Klose et al., 2012). Less abundant are cardiolipin (CL) and the lyso-lipid derivatives of the major glycerophospholipid classes with one fatty acid (FA) chain. We find that phosphatidylinositol (PI) is by far the most abundant lipid class in both WT and *ire1*Δ cells when cultivated in rich medium (Figure 3A and Supplementary Figure S3A). However, when cells are cultivated in synthetic medium, PI is much less abundant and found at levels comparable with phosphatidylcholine (PC) and phosphatidylethanolamine (PE) (Figure 3A and Supplementary Figure S3A). This is particularly relevant when studying the UPR: Ire1p has been identified as inositol-requiring enzyme (Nikawa and Yamashita, 1992), it is activated by inositol-depletion (Promlek et al., 2011), and sensitive to the stiffness of the ER membrane (Halbleib et al., 2017) thereby implying an important role of PI lipids. But not only PI, also the abundance of PE is affected by the choice the medium. PE is significantly less abundant in cells cultivated in rich versus synthetic medium (Figure 3A). Again, this is likely to affect the sensitivity and activity of the UPR as aberrant PE-to-PC ratios have been firmly implicated in chronic activation of the UPR in yeast, worms, and mammals (Fu et al., 2011; Thibault et al., 2012; Hou et al., 2014).

Although qualitatively consistent with previous reports (Ejsing et al., 2009; Klose et al., 2012; Surma et al., 2013; Casanovas et al., 2015) our observations also highlight an important caveat for the use of rich medium. Because YPD is not a defined medium (in contrast to the synthetic-defined SCD), its use will inevitably lead to inconsistencies with respect to the lipid composition. As a consequence, it is almost impossible to compare data coming from different laboratories using media from different suppliers or even media batches. It is also impossible to exclude a technical bias as contributing factor for seemingly divergent observations: different procedures for lipid extraction into an organic phase may affect the spectrum of lipids that can be detected, different modes of sample separation and/or detection [such as thin-layer chromatography (TLC), liquid chromatography coupled to mass spectrometry (LC-MS) or the shotgun lipidomics platform used for this study] might bias the detection of some lipids/lipid classes over others. Ideally, the use of internal standards should correct the bias from extraction and detection. Furthermore, only fully quantitative data expressed in absolute units (such as pmol or derived molar fractions) can be compared to each other, as any relative data depend on the experimental setup and reference points applicable only within a given experiment.

#### Impact of the Medium on Storage Lipids

The choice of the medium has a striking impact on the abundancies of storage lipids. Exponentially growing cells cultivated in rich medium store much less TAGs and EEs than those cultivated in synthetic medium (Figure 3A). The same trend is observed in *ire1*Δ cells (Supplementary Figure S3A). It is tempting to speculate that rapidly growing cells depend more heavily on the production of membrane lipids than relatively slow growing cells, which can ‘afford’ to store some lipids for future use. Clearly, the different growth rates in the two media (Figure 1 and Supplementary Figure S1) must be considered in light of the intricate connections between membrane biogenesis, lipid droplet formation, and the UPR (Gaspar et al., 2011; Stordeur et al., 2014; Casanovas et al., 2015). Our data underscore the importance to study the UPR under tightly controlled conditions.

#### Impact of the Medium on the Lipid Species Level Identifies Asymmetric Lipids

We wanted to know the impact of rich versus synthetic medium on lipid acyl chains and initially focused our attention on the profile of PA lipids (Figure 3B). Normally, in *S. cerevisiae*, the fatty acid composition of PA lipids is mostly limited to palmitic (C16:0), palmitoleic (C16:1) and oleic acid (C18:1) with low amounts of shorter fatty acids (Klose et al., 2012). However, the cells cultivated in rich medium exhibited a significantly higher abundance of PA lipids with shorter, saturated acyl chains, as evidenced by the level of PA lipids with a cumulative acyl chain length of C26, C28, and C30 (Figure 3B). A combined analysis of all MGLs with two fatty acyl chains (PA, PC, PE, PI, PS, DAG) further highlighted this general and significant shift toward shorter (Figure 3C) and more saturated lipid species (Figure 3D). Notably, identical trends can be observed for *ire1*Δ cells indicating that a low, basal activation of the UPR does not contribute to this global trend in all MGLs caused by the cultivation in different media (Supplementary Figures S3B–D).

We then studied the usage of different fatty acyl chains in PA, PC, and PI in cells cultivated in both rich and synthetic medium (Figure 3E) and also calculated the abundance of the different fatty acyl chains in all MGLs (with two fatty acyl chains) (Figure 3E). Consistent with previous reports, we find that the distribution of the fatty acyl chains differs between the individual glycerophospholipids classes (Figure 3E) (Ejsing et al., 2009; Klose et al., 2012; Casanovas et al., 2015). Strikingly, our data reveal that saturated, medium fatty acyl chains (C10, C12, and C14) are much more prominently found in cells cultivated in rich medium throughout all glycerophospholipid classes (Figure 3E). Notably, similar asymmetric lipids with two acyl chains differing substantially in length have only recently been found at high abundance in the lipidome of *Schizosaccharomyces japonicus* (Makarova et al., 2020).

In order to gain more insight in the molecular rules that govern the production of asymmetric lipids, we studied the pairing of fatty acyl chains in MGLs. We found an almost exclusive pairing of saturated, medium fatty acyl chains (C10:0 or C12:0) with longer saturated fatty acyl chains (C16:0 or C18:0) in *S. cerevisiae* (Figure 3F; left panel). In fact, we find virtually no pairing of unsaturated fatty acyl chain (C16:1 or C18:1) with medium fatty acyl chains (C10:0 or C12:0). Also when considering the relative abundance of the different fatty acyl chains (C16:0, C16:1 and C18:1 are most abundant), the same, remarkable selectivity for certain pairs of fatty acyl chains becomes clear: medium fatty acyl chains preferentially pair with saturated, but not with unsaturated fatty acyl chains (Figure 3F; right panel). YPD is not a fully defined medium and it may contain minor concentrations of short and medium chain fatty acids. In order to test if *S. cerevisiae* can synthesize asymmetric lipids from scratch, we analyzed the acyl chain pairing in cells cultivated in synthetic medium (Figure 3G). Again, we found a strong preferential pairing of saturated, medium fatty acyl chains with saturated, long fatty acid chains, but not with unsaturated ones (Figure 3G). Our data suggest that tight and evolutionary conserved rules underly the pairing of fatty acyl chains in these highly asymmetric lipids.

#### A Medium-Dependent Switch in the Species Distribution of Sphingolipids

Another remarkably specific impact of the medium can be observed in the profile of SLs for both WT (Figure 3H) and *ire1*Δ cells (Supplementary Figure S3E). The most abundant sphingolipid species of cells cultivated in rich medium is IPC 44:0,3 (with three hydroxylations) contributing to more than 50 mol% of all SLs, while IPC 44:0,4 (with four hydroxylations) is much less abundant. In contrast, IPC 44:0;4 is contributing to more than 40 mol% to the pool of SLs and is the most abundant SL species in SCD-cultured cells. The molecular underpinnings of this remarkable shift in the species composition and its physiological relevance remains to be studied in greater detail.

### The Impact of DTT and TM on the Lipidome of *S. cerevisiae* in Rich Medium

**FIGURE 4:**
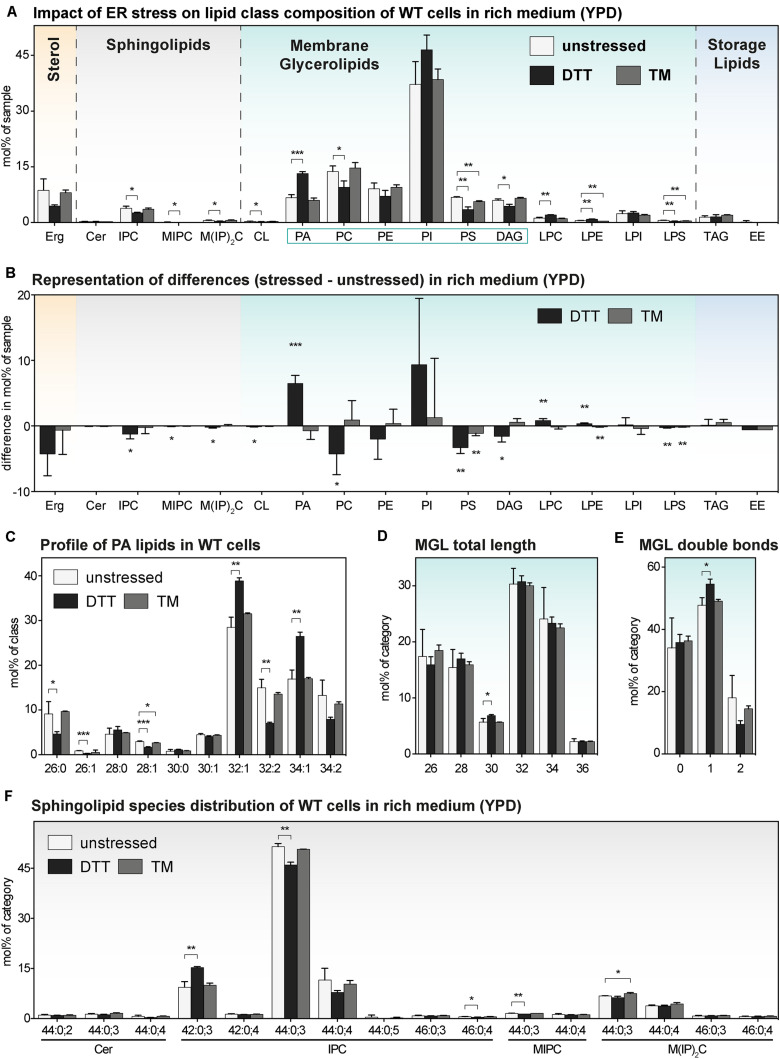
The impact of proteotoxic stress on the lipidome of *S. cerevisiae* WT in rich medium. A single colony of the BY4741 WT strain was used to inoculate a preculture in rich (YPD) medium. After overnight cultivation for 21 h, a fresh culture was inoculated to an OD_600_ of 0.1 and then cultivated to an OD_600_ of 0.80 ± 0.05. The cells were then either left untreated (white bars), stressed by the addition of either with 8 mM DTT (black bars) or 1.0 μg/ml TM (gray bars). After one additional hour of cultivation, 20 OD equivalents of these cells were harvested and analyzed by lipid mass spectrometry. **(A)** Lipid class composition in mol% of all quantified lipids in the sample organized by lipid categories. **(B)** The difference in lipid class abundance in stressed minus unstressed cells highlights the impact of DTT (black) and TM (gray) on the cellular lipid composition in rich medium. The difference in abundance in mol% was calculated by subtracting the abundance in unstressed cells from the abundance in either DTT- or TM-stressed cells under consideration of error propagation. **(C)** Profile of PA lipids in mol% of the class. **(D)** Total length of lipids in a sub-category of MGLs (PA, PC, PE, PI, PS, DAG). The total length is given as the sum of carbon atoms in both fatty acyl chains in mol% of this sub-category. **(E)** Total number double bonds in a sub-category of MGLs (PA, PC, PE, PI, PS, DAG) is given as the sum of double bonds in both acyl chains and represented in mol% of this sub-category. **(F)** Profile of sphingolipids in mol% of this category. The least abundant species are omitted for clarity. Each bar represents the average ± SD from *n* = 3 independent experiments. Statistical significance was tested by an unpaired two-tailed *t*-test using GraphPad Prism, *p < 0.05, **p < 0.01, ***p < 0.001.

**FIGURE 5:**
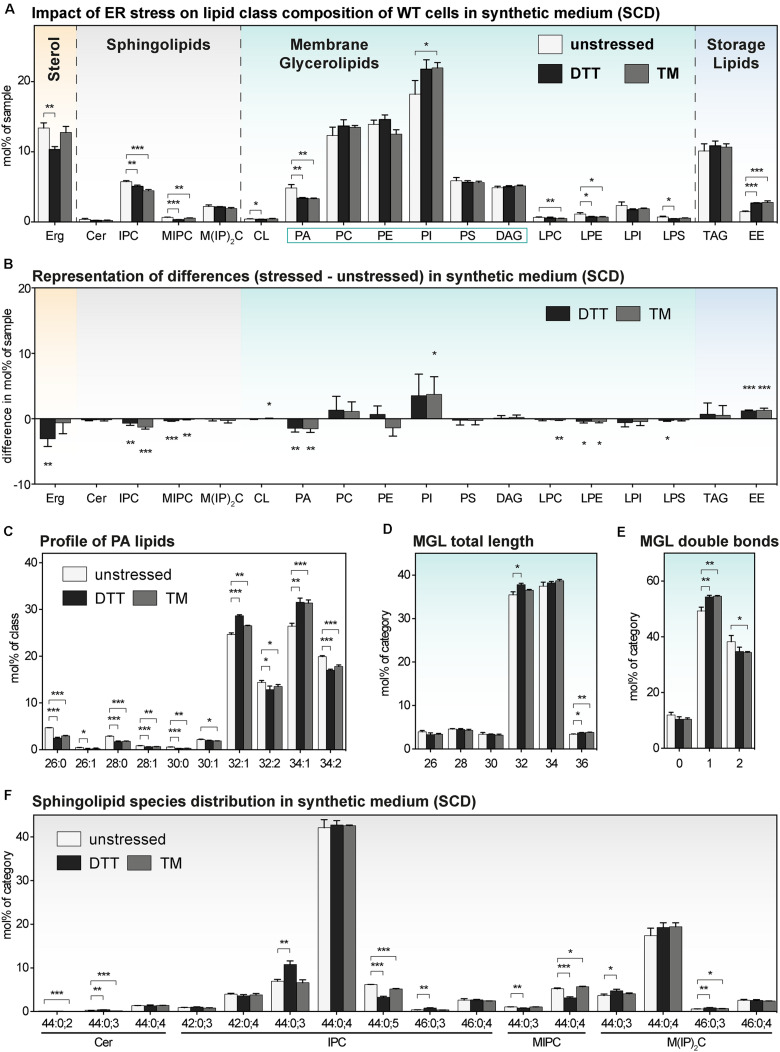
The impact of proteotoxic stress on the lipidome of*S. cerevisiae* WT cultivated in synthetic medium. A single colony of the BY4741 WT strain was used to inoculate a preculture in synthetic medium (SCD). After overnight cultivation for 21 h, a fresh culture was inoculated to an OD_600_ of 0.1 and then cultivated to an OD_600_ of 0.80 ± 0.05. The cells were then either left untreated (white bars), stressed by the addition of either with 8 mM DTT (black bars) or 1.0 μg/ml TM (gray bars). After one additional hour of cultivation, 20 OD equivalents of these cells were harvested and analyzed by lipid mass spectrometry. **(A)** Lipid class composition in mol% of all quantified lipids in the sample organized by lipid categories. **(B)** The difference in lipid class abundance in stressed minus unstressed cells highlights the impact of DTT (black) and TM (gray) on the cellular lipid composition in synthetic medium. The difference in abundance in mol% was calculated by subtracting the abundance in unstressed cells from the abundance in either DTT- or TM-stressed cells under consideration of error propagation. **(C)** Profile of PA lipids in mol% of the class. **(D)** Total length of lipids in a sub-category of MGLs (PA, PC, PE, PI, PS, DAG). The total length is given as the sum of carbon atoms in both fatty acyl chains in mol% of this sub-category. **(E)** Total number double bonds in a sub-category of MGLs (PA, PC, PE, PI, PS, DAG) is given as the sum of double bonds in both acyl chains and represented in mol% of this sub-category. **(F)** Profile of sphingolipids in mol% of this category. The least abundant species are omitted for clarity. Each bar represents the average ± SD from *n* = 3 independent experiments. Statistical significance was tested by an unpaired two-tailed *t*-test using GraphPad Prism, *p < 0.05, **p < 0.01, ***p < 0.001.

We wanted to study the impact of DTT and TM on the lipid composition of cells cultivated in both rich medium (Figure 4) and synthetic medium (Figure 5) and focus our attention on an early time point after 1 h of treatment. As important controls, we also determined the lipid compositions of stressed and unstressed *ire1*Δ cells in both media to test a possible contribution of UPR to changes of the lipidome (Supplementary Figures S4, S5).

Consistent with our PCA analysis (Figure 2B), we find that the TM-induced stress has barely any impact on the lipid composition of WT cells (Figure 4A). This lack-of-impact is best evident when representing difference of abundance between stressed and unstressed cells for each lipid class (Figure 4B). There is also barely any impact of TM on the profile of PA lipids (Figure 4C), the length distribution of the fatty acyl chains (Figure 4D), the degree of saturation (Figure 4E), and the species composition of SLs (Figure 4F). Notably, this is true for both for WT and *ire1*Δ cells (Supplementary Figures S4A–F). These data suggest TM can be used as proteotoxic drug in YPD-cultivated cells without severely affecting the cellular lipid composition.

In contrast to that, DTT at the given concentration has a significant impact on the lipidome of YPD-cultured cells (Figure 4A), which is most apparent when plotting the difference of lipid class abundance between stressed and unstressed cells (Figure 4B). Clearly, treating the cells for only 1 h with DTT is sufficient to cause a significant and substantial increase of PA in the stressed cells. Given that PA lipids are important signaling lipids involved in regulating membrane biogenesis (Loewen, 2004; Schuck et al., 2009; Henry et al., 2012; Hofbauer et al., 2018), a two-fold increase of the cellular PA level is likely to have broad impact on lipid metabolism and the cellular transcriptome. Intriguingly, treating the cells with DTT induces also a shift in the profile of PA lipids toward a higher average acyl chain length and more unsaturation for both WT (Figure 4C) and *ire1*Δ cells (Supplementary Figure S4C). While these observations suggest that DTT affects PA lipids either directly (by affecting fatty acid metabolism) or indirectly (by its impact on cellular growth or other means; Supplementary Figure S1A), these trends are not pronounced in the wider group of MGLs with two fatty acyl chains – neither for WT nor *ire1*Δ cells (Figures 4D,E and Supplementary Figures S4D,E). Based on its position in the lipid metabolic network (Henry et al., 2012; Ernst et al., 2016), we speculate that PA is a class of ‘early-responding’ lipids, which change most readily upon an environmental perturbation, while other MGLs are affected only after prolonged periods of stress.

We also find some impact of ER stress on the profile of SLs of WT and *ire1*Δ cells (Figure 4F and Supplementary Figure S4F), but these changes are relatively small. The overall similarity of the lipidomic changes observed for WT and *ire1*Δ cells upon DTT treatments, suggest that most DTT impacts on lipid metabolism are independently of UPR-signaling, at least under the given conditions (Figure 4 and Supplementary Figure S4).

In summary, we find that even a short cultivation in the presence of DTT induces substantial changes of the cellular lipidome.

### The Impact of DTT and TM on the Lipidome of *S. cerevisiae* in Synthetic Medium

We wanted to know the impact of DTT and TM on the lipid composition of *S. cerevisiae* cultivated in synthetic medium. We therefore determined the lipidomes of both stressed and unstressed WT and *ire1*Δ cells (Figure 5 and Supplementary Figure S5). Overall, we find an almost identical lipid class composition for cells stressed either with DTT or TM compared to untreated control cells with only minor changes in the abundance of several lipid classes (Figures 5A,B and Supplementary Figures S5A,B). While the changes between stressed and unstressed cells are significant, the difference in abundance seems rather moderate (Figure 5B). For example, the ∼30% lower level of PA in both DTT- and TM-stressed cells (Figure 5A), has most probably a lower impact on cellular physiology than the two-fold increase of PA in DTT-treated cells in rich medium (Figure 4A). Other significant, moderate changes are observed for several SLs, Erg, and EEs (Figure 5A). We doubt that these lipidome changes are substantial enough to impact on the activity of the UPR via a membrane-based mechanism. This speculation is supported by the observation that proteotoxic stress clearly dominates over membrane-based stress when cells are treated with either DTT or TM for 1 h or less (Promlek et al., 2011).

Beyond the overall very similar lipid class distribution of stressed and unstressed cells cultivated in synthetic medium, we find significant, but minor stress-induced changes in the PA species composition (Figure 5C), a similar total fatty acyl chain length in MGLs (Figure 5D), a mildly affected degree of lipid saturation (Figure 5E), and a largely similar profile of SLs (Figure 5F). Again, the lipidomes observed for WT and *ire1*Δ cells are extremely similar. In summary, we find, somewhat surprisingly, that the cellular lipidome is quite ‘resistant’ to perturbations from DTT- and TM-treatments when cells are treated for 1 h in synthetic medium.

Our lipidomic survey of stressed and unstressed *S. cerevisiae* reveals that TM treatments for 1 h are suitable in both rich and synthetic medium to activate the UPR via proteotoxic stress with only little interference from a perturbed lipid composition. However, more precautions should be taken when using DTT.

## Discussion

We have performed a systematic, quantitative analysis of the lipidomic changes associated with acute ER stress caused by the proteotoxic agents DTT and TM. Given the central relevance of the UPR for cellular homeostasis and the adverse effects associated with chronic UPR activation, it is crucial to better understand the signals that underlie prolonged and chronic activation of the UPR in the future.

### Recommendations for Studying UPR-Activating Signals

Using *S. cerevisiae* as a model, we highlight the importance of tightly controlled cultivation conditions and quantitative lipid analyses for a more holistic approach toward understanding the interplay of UPR-activating signals. We show that (1) the proteotoxic drugs DTT and TM impair cellular growth, thereby confirming previous observations (Pincus et al., 2010), (2) DTT causes within 1 h of treatment substantial changes of the lipidome of YPD-cultured cells, (3) DTT and TM have only a minor impact on the lipidome of SCD-cultured cells, (4) unstressed WT and *ire1*Δ cells feature virtually identical lipidomes under the tested conditions, thereby suggesting that basal, low-level UPR signaling (or lack of thereof) does not substantially affect the cellular lipid composition. Overall, our data are consistent with the general assumption that TM predominantly causes proteotoxic stress, at least within the first hour of treatment (Promlek et al., 2011). The rapid, DTT-dependent remodeling of the lipidome observed in YPD-cultured cells and the strongly impaired growth of stressed cultures, however, may serve as a warning to carefully interpret data derived from acutely stressed cells. We like to stress that we investigated only the impact of a single concentration for each drug in both media on the cellular lipid composition. It is possible that TM, when used at higher concentrations, might have a more severe impact on the cellular lipid composition. Nevertheless, for studying the UPR and its response to proteotoxic signals with little to no interference from a perturbed lipid metabolism, we suggest (1) the use of defined SCD over ill-defined YPD, (2) the use of TM over DTT, because of its more specific impact on protein folding in the ER, and (3) applying TM or DTT stress for a maximum of 1 h.

### Membrane Biogenesis in Rapidly Growing Cells – Competition for Lipid Metabolites

Our systematic lipidomic analyses of WT and *ire1*Δ cells in two different media provides insights into the orchestration of membrane biogenesis by rapidly growing, eukaryotic cells. Even though rich medium provides a rich supply of nutrients, cells cultivated in this medium do not accumulate substantial amounts of storage lipids during the exponential growth phase (Figure 3A). Cells cultivated in synthetic medium, however, accumulate a five times higher level of neutral lipids (TAGs + EEs) although the medium is not as rich (Figure 3A). We speculate that these marked differences are at least partly due to the growth rate, which is substantially higher for YPD-cultured cells. This interpretation is consistent with the previous observation that storage lipids are dynamically regulated in a growth-stage dependent fashion (Klose et al., 2012; Kohlwein et al., 2013; Casanovas et al., 2015) and suggests an increased flux of fatty acids into membrane lipids in rapidly growing cells. In fact, the lipid phenotypes associated with a genetically disrupted fatty acid desaturation are more pronounced in YPD-cultured cells (Surma et al., 2013). The global role of the cellular growth rate on lipid metabolism remains to be established in the future.

Along these lines, we also find that the ratio of SLs to MGLs is low in rapidly growing, YPD-cultured cells, but higher in slow-growing SCD-cultured cells (Figure 3A). While it is clear that many distinct regulatory circuits of the cell are affected by the composition of the medium, we speculate that the low SL-to-MGL ratio reflects an increased global rate of the lipid biosynthesis in the ER and -as a consequence- a low ratio of plasma membrane-to-ER abundance. Notably, our finding that WT and *ire1*Δ cells feature almost identical lipidomes (Supplementary Figure S3) would suggest that ER abundance is controlled independently of the UPR in this case. This is consistent with previous findings that ER proliferation can be uncoupled from the UPR via the Opi1 and Ino2/Ino4 regulatory circuit (Loewen, 2004; Schuck et al., 2009; Henry et al., 2012). Opi1 binding to PA lipids at the ER membrane (Loewen, 2004; Hofbauer et al., 2018) de-represses Ino2/Ino4-regulated genes involved in MGL biosynthesis and tip the balance from storing neutral lipids toward membrane proliferation. In fact, we find an increased level of PA lipids in YPD-cultured cells (Figure 3A and Supplementary Figure S3A). Our findings fuel that idea that the flux of fatty acids into either membrane or storage lipids is affected by the cellular growth rate, which shall investigated by dedicated experiments in the future.

### Revealing Acyl Chain Selectivity in Saturated, Asymmetric Lipids

We were surprised by the high content of saturated lipids in YPD-grown cells (Figures 3D,E), which differs from previous studies (Klose et al., 2012; Surma et al., 2013; Casanovas et al., 2015) and which resembles lipid phenotypes observed only in genetically modified *S. cerevisiae* with a disrupted fatty acid desaturation (Pineau et al., 2009; Surma et al., 2013; Ballweg and Ernst, 2017; Budin et al., 2018). The majority of these saturated lipids contain a medium fatty acyl chain (C10:0 or C12:0) paired with a second, long fatty acyl chain (C16:0 or C18:0). Such asymmetric, saturated lipids at even higher abundancies have been recently identified in *Schizosaccharomyces japonicus* (Makarova et al., 2020). The length difference of the two acyl chains may allow for an interdigitation of the acyl chains, which increases lipid packing, whilst maintaining a fluid bilayer (Xu and Huang, 1987; Schram and Thompson, 1995; Makarova et al., 2020). This way, the asymmetric lipids can provide an alternative handle to balance competing demands in the homeostasis of physicochemical membrane properties, e.g., by maintaining membrane barrier function whilst increasing membrane fluidity (Lande et al., 1995; Schram and Thompson, 1995; Radanović et al., 2018). This may become very relevant under conditions when medium-chain fatty acids accumulate in the cell and/or when the desaturation of long-chain fatty acids via the fatty acid desaturase Ole1 becomes limiting (Stukey et al., 1989; Ballweg and Ernst, 2017). How precisely saturated, asymmetric lipids provide a feedback to the production of unsaturated fatty acid via the lipid saturation sensor Mga2 (Covino et al., 2016; Ballweg et al., 2020) is an interesting question for the future. Our finding that asymmetric, saturated lipids can be observed at substantial levels in *S. cerevisiae* suggests that such lipids may play a much wider role than previously anticipated.

The machineries and mechanisms mediating a finely tuned production of asymmetric lipids in *S. cerevisiae* and other fungi are still unknown, but tracking the origin and fate of medium chain fatty acids might help identifying them. Our finding that saturated, asymmetric lipids are produced even upon cultivation in fatty acid-free SCD medium (Figure 3G) suggests that at least a significant portion of the esterified medium chain fatty acids originate from *de novo* biosynthesis. The fungal fatty acid synthase produces fatty acids of different lengths determined by the cellular ratio of acetyl-CoA to malonyl-CoA, which are used for priming and fatty acid elongation (Sumper et al., 1969; Okuyama et al., 1979; Heil et al., 2019). It is possible that a different product spectrum of the fatty acid synthase contributes to the production and abundance of asymmetric lipids in *S. cerevisiae*. Intriguingly, a hyper-active mutation in the rate-limiting enzyme for fatty acid biosynthesis leads to an increased production of malony-CoA and increased average fatty acyl chain length in glycerophospholipids (Hofbauer et al., 2014). In line with previous reports (Makarova et al., 2020), we therefore propose that the profile of *de novo* synthesized fatty acids is a major determinant for the abundance of saturated, asymmetric lipids.

Our finding that saturated, medium fatty acyl chain pair almost exclusive with saturated, but not with the more abundant unsaturated fatty acyl chains (Figures 3E–G), reveals a remarkable, inherent selectivity in the biosynthesis and metabolism of asymmetric lipids. It will be intriguing to learn about the processes that contribute to this selectivity and to dissect the contribution of fatty acid biosynthesis and activation, acyl transferases, phospholipases, and substrate channeling (Henry et al., 2012; Ernst et al., 2016; Patton-Vogt and de Kroon, 2020). Because the key enzymes of lipid metabolism and the principle mechanisms of membrane adaptivity are conserved throughout evolution (Henry et al., 2012; Ernst et al., 2016), it will be intriguing to test if similarly specific mechanisms of acyl chain pairing are at work in organisms from bacteria to humans.

## Conclusion

The UPR has been implicated in a wide array of pathologies and is gaining increasing attention as a potential drug target (Hetz et al., 2019). While the fatal consequences of prolonged ER stress have been intensively studied (Tabas and Ron, 2011), the molecular events that cause chronic UPR activation remain poorly characterized. In the future, it will be crucial to develop new tools and assays to better understand the signals that perpetuate ER stress. Only quantitative information on the ER load with unfolded proteins and on the composition of the ER membrane during acute and prolonged ER stress can unambiguously establish the relative importance of signals from the ER lumen and the ER membrane. As a first step in this direction, we have studied the impact of different media and two UPR-activating, proteotoxic drugs on the cellular lipid composition. Our data will provide an important reference point for future endeavors, and has already proven useful for highlighting possible connections between the cellular growth rate, proteotoxic ER stress, and lipid metabolism. Our finding that *S. cerevisiae* produces asymmetric lipids with two saturated acyl chains of different lengths provides evidence for a remarkable specificity in paring of saturated fatty acyl chains.

## Data Availability Statement

All datasets presented in this study are included in the article/Supplementary Material.

## Author Contributions

RE conceived and designed the experiments. JR, CM, KV, MS, and CK performed the experiments. JR, CM, KV, and TR analyzed the data. JR, MS, and CK contributed reagents, materials, and analysis tools. JR, CM, TR, and RE wrote the manuscript. All authors contributed to the article and approved the submitted version.

## Conflict of Interest

MS and CK were employed by the company Lipotype GmbH, Dresden. The remaining authors declare that the research was conducted in the absence of any other commercial or financial relationships that could be construed as a potential conflict of interest.

## References

[B1] Adams C. J. Kopp M. C. Larburu N. Nowak P. R. Ali M. M. U. (2019). Structure and molecular mechanism of ER stress signaling by the unfolded protein response signal activator IRE1. *Front. Mol. Biosci.* 6:11. 10.3389/fmolb.2019.00011 30931312 PMC6423427

[B2] Aviram N. Schuldiner M. (2017). Targeting and translocation of proteins to the endoplasmic reticulum at a glance. *J. Cell Sci.* 130 4079–4085. 10.1242/jcs.204396 29246967

[B3] Azim M. Surani H. (1979). Glycoprotein synthesis and inhibition of glycosylation by tunicamycin in preimplantation mouse embryos: compaction and trophoblast adhesion. *Cell* 18 217–227. 10.1016/0092-8674(79)90370-2 509524

[B4] Ballweg S. Ernst R. (2017). Control of membrane fluidity: the OLE pathway in focus. *Biol. Chem.* 398 215–228. 10.1515/hsz-2016-0277 27787227

[B5] Ballweg S. Sezgin E. Doktorova M. Covino R. Reinhard J. Wunnicke D. (2020). Regulation of lipid saturation without sensing membrane fluidity. *Nat. Commun.* 11:756. 10.1038/s41467-020-14528-1 32029718 PMC7005026

[B6] Bernales S. McDonald K. L. Walter P. (2006). Autophagy counterbalances endoplasmic reticulum expansion during the unfolded protein response. *PLoS Biol.* 4:e423. 10.1371/journal.pbio.0040423 17132049 PMC1661684

[B7] Bernales S. Schuck S. Walter P. (2007). ER-Phagy: selective autophagy of the endoplasmic reticulum. *Autophagy* 3 285–287. 10.4161/auto.3930 17351330

[B8] Bigay J. Antonny B. (2012). Curvature, lipid packing, and electrostatics of membrane organelles: defining cellular territories in determining specificity. *Dev. Cell* 23 886–895. 10.1016/j.devcel.2012.10.009 23153485

[B9] Budin I. de Rond T. Chen Y. Chan L. J. G. Petzold C. J. Keasling J. D. (2018). Viscous control of cellular respiration by membrane lipid composition. *Science* 362 1186–1189. 10.1126/science.aat7925 30361388

[B10] Casanovas A. Sprenger R. R. Tarasov K. Ruckerbauer D. E. Hannibal-Bach H. K. Zanghellini J. (2015). Quantitative analysis of proteome and lipidome dynamics reveals functional regulation of global lipid metabolism. *Chem. Biol.* 22 412–425. 10.1016/j.chembiol.2015.02.007 25794437

[B11] Covino R. Ballweg S. Stordeur C. Michaelis J. B. Puth K. Wernig F. (2016). A eukaryotic sensor for membrane lipid saturation. *Mol. Cell* 63 49–59. 10.1016/j.molcel.2016.05.015 27320200

[B12] Covino R. Hummer G. Ernst R. (2018). Integrated functions of membrane property sensors and a hidden side of the unfolded protein response. *Mol. Cell* 71 458–467. 10.1016/j.molcel.2018.07.019 30075144

[B13] Cox J. S. Shamu C. E. Walter P. (1993). Transcriptional induction of genes encoding endoplasmic reticulum resident proteins requires a transmembrane protein kinase. *Cell* 73 1197–1206. 10.1016/0092-8674(93)90648-A 8513503

[B14] Cox J. S. Walter P. (1996). A novel mechanism for regulating activity of a transcription factor that controls the unfolded protein response. *Cell* 87 391–404. 10.1016/s0092-8674(00)81360-4 8898193

[B15] De Kroon A. I. P. M. Rijken P. J. De Smet C. H. (2013). Checks and balances in membrane phospholipid class and acyl chain homeostasis, the yeast perspective. *Prog. Lipid Res.* 52 374–394. 10.1016/j.plipres.2013.04.006 23631861

[B16] Dymond J. S. (2013). *Saccharomyces cerevisiae* growth media. *Methods Enzymol.* 533 191–204. 10.1016/B978-0-12-420067-8.00012-X 24182924

[B17] Ejsing C. S. Sampaio J. L. Surendranath V. Duchoslav E. Ekroos K. Klemm R. W. (2009). Global analysis of the yeast lipidome by quantitative shotgun mass spectrometry. *Proc. Natl. Acad. Sci. U.S.A.* 106 2136–2141. 10.1073/pnas.0811700106 19174513 PMC2650121

[B18] Ernst R. Ballweg S. Levental I. (2018). Cellular mechanisms of physicochemical membrane homeostasis. *Curr. Opin. Cell Biol.* 53 44–51. 10.1016/j.ceb.2018.04.013 29787971 PMC6131038

[B19] Ernst R. Ejsing C. S. Antonny B. (2016). Homeoviscous adaptation and the regulation of membrane lipids. *J. Mol. Biol.* 428 4776–4791. 10.1016/j.jmb.2016.08.013 27534816

[B20] Fonseca S. G. Burcin M. Gromada J. Urano F. (2009). Endoplasmic reticulum stress in β-cells and development of diabetes. *Curr. Opin. Pharmacol.* 9 763–770. 10.1016/j.coph.2009.07.003 19665428 PMC2787771

[B21] Fu S. Yang L. Li P. Hofmann O. Dicker L. Hide W. (2011). Aberrant lipid metabolism disrupts calcium homeostasis causing liver endoplasmic reticulum stress in obesity. *Nature* 473 528–531. 10.1038/nature09968 21532591 PMC3102791

[B22] Fun X. H. Thibault G. (2020). Lipid bilayer stress and proteotoxic stress-induced unfolded protein response deploy divergent transcriptional and non-transcriptional programmes. *Biochim. Biophys. Acta* 1865:158449. 10.1016/j.bbalip.2019.04.009 31028913

[B23] Gaspar M. L. Hofbauer H. F. Kohlwein S. D. Henry S. A. (2011). Coordination of storage lipid synthesis and membrane biogenesis: evidence for cross-talk between triacylglycerol metabolism and phosphatidylinositol synthesis. *J. Biol. Chem.* 286 1696–1708. 10.1074/jbc.M110.172296 20972264 PMC3023464

[B24] Goder V. Alanis-Dominguez E. Bustamante-Sequeiros M. (2020). Lipids and their (un)known effects on ER-associated protein degradation (ERAD). *Biochim. Biophys. Acta* 1865:158488. 10.1016/j.bbalip.2019.06.014 31233887

[B25] Halbleib K. Pesek K. Covino R. Hofbauer H. F. Wunnicke D. Hänelt I. (2017). Activation of the unfolded protein response by lipid bilayer stress. *Mol. Cell* 67 673–684.e8. 10.1016/j.molcel.2017.06.012 28689662

[B26] Han X. (2016). Lipidomics for studying metabolism. *Nat. Rev. Endocrinol.* 12 668–679. 10.1038/nrendo.2016.98 27469345

[B27] Hannich J. T. Umebayashi K. Riezman H. (2011). Distribution and functions of sterols and sphingolipids. *Cold Spring Harb. Perspect. Biol.* 3:a004762. 10.1101/cshperspect.a004762 21454248 PMC3101845

[B28] Hanscho M. Ruckerbauer D. E. Chauhan N. Hofbauer H. F. Krahulec S. Nidetzky B. (2012). Nutritional requirements of the BY series of *Saccharomyces cerevisiae* strains for optimum growth. *FEMS Yeast Res.* 12 796–808. 10.1111/j.1567-1364.2012.00830.x 22780918

[B29] Harayama T. Riezman H. (2018). Understanding the diversity of membrane lipid composition. *Nat. Rev. Mol. Cell Biol.* 19 281–296. 10.1038/nrm.2017.138 29410529

[B30] Heil C. S. Wehrheim S. S. Paithankar K. S. Grininger M. (2019). Fatty acid biosynthesis: chain-length regulation and control. *ChemBioChem* 20 2298–2321. 10.1002/cbic.201800809 30908841

[B31] Henry S. A. Kohlwein S. D. Carman G. M. (2012). Metabolism and regulation of glycerolipids in the yeast Saccharomyces cerevisiae. *Genetics* 190 317–349. 10.1534/genetics.111.130286 22345606 PMC3276621

[B32] Herzog R. Schuhmann K. Schwudke D. Sampaio J. L. Bornstein S. R. Schroeder M. (2012). Lipidxplorer: a software for consensual cross-platform lipidomics. *PLoS One* 7:e29851. 10.1371/journal.pone.0029851 22272252 PMC3260173

[B33] Herzog R. Schwudke D. Schuhmann K. Sampaio J. L. Bornstein S. R. Schroeder M. (2011). A novel informatics concept for high-throughput shotgun lipidomics based on the molecular fragmentation query language. *Genome Biol.* 12:R8. 10.1186/gb-2011-12-1-r8 21247462 PMC3091306

[B34] Hetz C. Axten J. M. Patterson J. B. (2019). Pharmacological targeting of the unfolded protein response for disease intervention. *Nat. Chem. Biol.* 15 764–775. 10.1038/s41589-019-0326-2 31320759

[B35] Hofbauer H. F. Gecht M. Fischer S. C. Seybert A. Frangakis A. S. Stelzer E. H. K. (2018). The molecular recognition of phosphatidic acid by an amphipathic helix in Opi1. *J. Cell Biol.* 217 3109–3126. 10.1083/jcb.201802027 29941475 PMC6122994

[B36] Hofbauer H. F. Schopf F. H. Schleifer H. Knittelfelder O. L. Pieber B. Rechberger G. N. (2014). Regulation of gene expression through a transcriptional repressor that senses acyl-chain length in membrane phospholipids. *Dev. Cell* 29 729–739. 10.1016/j.devcel.2014.04.025 24960695 PMC4070385

[B37] Hou N. S. Gutschmidt A. Choi D. Y. Pather K. Shi X. Watts J. L. (2014). Activation of the endoplasmic reticulum unfolded protein response by lipid disequilibrium without disturbed proteostasis *in vivo*. *Proc. Natl. Acad. Sci. U.S.A.* 111 E2271–E2280. 10.1073/pnas.1318262111 24843123 PMC4050548

[B38] Hsu J. W. Tang P. H. Wang I. H. Liu C. L. Chen W. H. Tsai P. C. (2016). Unfolded protein response regulates yeast small GTPase Arl1p activation at late Golgi via phosphorylation of Arf GEF Syt1p. *Proc. Natl. Acad. Sci. U.S.A.* 113 E1683–E1690. 10.1073/pnas.1518260113 26966233 PMC4812715

[B39] Jonikas M. C. Collins S. R. Denic V. Oh E. Quan E. M. Schmid V. (2009). Comprehensive characterization of genes required for protein folding in the endoplasmic reticulum. *Science* 323 1693–1697. 10.1126/science.1167983 19325107 PMC2877488

[B40] Karagöz G. E. Acosta-Alvear D. Nguyen H. T. Lee C. P. Chu F. Walter P. (2017). An unfolded protein-induced conformational switch activates mammalian IRE1. *eLife* 6:e30700. 10.7554/eLife.30700 28971800 PMC5699868

[B41] Kaufman R. J. (2002). Orchestrating the unfolded protein response in health and disease. *J. Clin. Invest.* 110 1389–1398. 10.1172/jci16886 12438434 PMC151822

[B42] Kawahara T. Yanagi H. Yura T. Mori K. (1997). Endoplasmic reticulum stress-induced mRNA splicing permits synthesis of transcription factor Hac1p/Ern4p that activates the unfolded protein response. *Mol. Biol. Cell* 8 1845–1862. 10.1091/mbc.8.10.1845 9348528 PMC25627

[B43] Kimata Y. Ishiwata-Kimata Y. Ito T. Hirata A. Suzuki T. Oikawa D. (2007). Two regulatory steps of ER-stress sensor Ire1 involving its cluster formation and interaction with unfolded proteins. *J. Cell Biol.* 179 75–86. 10.1083/jcb.200704166 17923530 PMC2064738

[B44] Kimata Y. Kohno K. (2011). Endoplasmic reticulum stress-sensing mechanisms in yeast and mammalian cells. *Curr. Opin. Cell Biol.* 23 135–142. 10.1016/j.ceb.2010.10.008 21093243

[B45] Klemm R. W. Ejsing C. S. Surma M. A. Kaiser H. J. Gerl M. J. Sampaio J. L. (2009). Segregation of sphingolipids and sterols during formation of secretory vesicles at the trans-Golgi network. *J. Cell Biol.* 185 601–612. 10.1083/jcb.200901145 19433450 PMC2711577

[B46] Klose C. Surma M. A. Gerl M. J. Meyenhofer F. Shevchenko A. Simons K. (2012). Flexibility of a eukaryotic lipidome - insights from yeast lipidomics. *PLoS One* 7:e35063. 10.1371/journal.pone.0035063 22529973 PMC3329542

[B47] Kohlwein S. D. Veenhuis M. van der Klei I. J. (2013). Lipid droplets and peroxisomes: key players in cellular lipid homeostasis or a matter of fat-store’em up or burn’em down. *Genetics* 193 1–50. 10.1534/genetics.112.143362 23275493 PMC3527239

[B48] Korennykh A. V. Egea P. F. Korostelev A. A. Finer-Moore J. Zhang C. Shokat K. M. (2009). The unfolded protein response signals through high-order assembly of Ire1. *Nature* 457 687–693. 10.1038/nature07661 19079236 PMC2846394

[B49] Lande M. B. Donovan J. M. Zeidel M. L. (1995). The relationship between membrane fluidity and permeabilities to water, solutes, ammonia, and protons. *J. Gen. Physiol.* 106 67–84. 10.1085/jgp.106.1.67 7494139 PMC2229255

[B50] Lee A. G. (2004). How lipids affect the activities of integral membrane proteins. *Biochim. Biophys. Acta* 1666 62–87. 10.1016/j.bbamem.2004.05.012 15519309

[B51] Levental I. Lingwood D. Grzybek M. Coskun Ü. Simons K. (2010). Palmitoylation regulates raft affinity for the majority of integral raft proteins. *Proc. Natl. Acad. Sci. U.S.A.* 107 22050–22054. 10.1073/pnas.1016184107 21131568 PMC3009825

[B52] Levental K. R. Malmberg E. Symons J. L. Fan Y. Y. Chapkin R. S. Ernst R. (2020). Lipidomic and biophysical homeostasis of mammalian membranes counteracts dietary lipid perturbations to maintain cellular fitness. *Nat. Commun.* 11:1339. 10.1038/s41467-020-15203-1 32165635 PMC7067841

[B53] Liebisch G. Binder M. Schifferer R. Langmann T. Schulz B. Schmitz G. (2006). High throughput quantification of cholesterol and cholesteryl ester by electrospray ionization tandem mass spectrometry (ESI-MS/MS). *Biochim. Biophys. Acta* 1761 121–128. 10.1016/j.bbalip.2005.12.007 16458590

[B54] Loewen C. J. R. (2004). Phospholipid metabolism regulated by a transcription factor sensing phosphatidic acid. *Science* 304 1644–1647. 10.1126/science.1096083 15192221

[B55] Lorent J. H. Diaz-Rohrer B. Lin X. Spring K. Gorfe A. A. Levental K. R. (2017). Structural determinants and functional consequences of protein affinity for membrane rafts. *Nat. Commun.* 8:1219. 10.1038/s41467-017-01328-3 29089556 PMC5663905

[B56] Makarova M. Peter M. Balogh G. Glatz A. MacRae J. I. Lopez Mora N. (2020). Delineating the rules for structural adaptation of membrane-associated proteins to evolutionary changes in membrane lipidome. *Curr. Biol.* 30 367–380.e8. 10.1016/j.cub.2019.11.043 31956022 PMC6997885

[B57] Mesecke N. Terziyska N. Kozany C. Baumann F. Neupert W. Hell K. (2005). A disulfide relay system in the intermembrane space of mitochondria that mediates protein import. *Cell* 121 1059–1069. 10.1016/j.cell.2005.04.011 15989955

[B58] Mori K. Kawahara T. Yoshida H. Yanagi H. Yura T. (1996). Signalling from endoplasmic reticulum to nucleus: transcription factor with a basic-leucine zipper motif is required for the unfolded protein-response pathway. *Genes Cells* 1 803–817. 10.1046/j.1365-2443.1996.d01-274.x 9077435

[B59] Nikawa J. Yamashita S. (1992). IRE1 encodes a putative protein kinase containing a membrane-spanning domain and is required for inositol phototrophy in Saccharomyces cerevisiae. *Mol. Microbiol.* 6 1441–1446. 10.1111/j.1365-2958.1992.tb00864.x 1625574

[B60] Novick P. Field C. Schekman R. (1980). Identification of 23 complementation groups required for post-translational events in the yeast secretory pathway. *Cell* 21 205–215. 10.1016/0092-8674(80)90128-2 6996832

[B61] Okuyama H. Saito M. Joshi V. C. Gunsberg S. Wakil S. J. (1979). Regulation by temperature of the chain length of fatty acids in yeast. *J. Biol. Chem.* 254 12281–12284. 387785

[B62] Patton-Vogt J. de Kroon A. I. P. M. (2020). Phospholipid turnover and acyl chain remodeling in the yeast ER. *Biochim. Biophys. Acta* 1865:158462. 10.1016/j.bbalip.2019.05.006 31146038 PMC10716787

[B63] Phillips R. Ursell T. Wiggins P. Sens P. (2009). Emerging roles for lipids in shaping membrane-protein function. *Nature* 459 379–385. 10.1038/nature08147 19458714 PMC3169427

[B64] Pincus D. Chevalier M. W. Aragón T. van Anken E. Vidal S. E. El-Samad H. (2010). BiP binding to the ER-stress sensor Ire1 tunes the homeostatic behavior of the unfolded protein response. *PLoS Biol.* 8:e1000415. 10.1371/journal.pbio.1000415 20625545 PMC2897766

[B65] Pineau L. Colas J. Dupont S. Beney L. Fleurat-Lessard P. Berjeaud J. M. (2009). Lipid-induced ER stress: synergistic effects of sterols and saturated fatty acids. *Traffic* 10 673–690. 10.1111/j.1600-0854.2009.00903.x 19302420

[B66] Preissler S. Ron D. (2019). Early events in the endoplasmic reticulum unfolded protein response. *Cold Spring Harb. Perspect. Biol.* 11:a033894. 10.1101/cshperspect.a033894 30396883 PMC6442202

[B67] Promlek T. Ishiwata-Kimata Y. Shido M. Sakuramoto M. Kohno K. Kimata Y. (2011). Membrane aberrancy and unfolded proteins activate the endoplasmic reticulum stress sensor Ire1 in different ways. *Mol. Biol. Cell* 22 3520–3532. 10.1091/mbc.E11-04-0295 21775630 PMC3172275

[B68] Radanović T. Reinhard J. Ballweg S. Pesek K. Ernst R. (2018). An emerging group of membrane property sensors controls the physical state of organellar membranes to maintain their identity. *Bioessays* 40:e1700250. 10.1002/bies.201700250 29574931

[B69] Schram V. Thompson T. E. (1995). Interdigitation does not affect translational diffusion of lipids in liquid crystalline bilayers. *Biophys. J.* 69 2517–2520. 10.1016/s0006-3495(95)80122-0 8599658 PMC1236489

[B70] Schuck S. Prinz W. A. Thorn K. S. Voss C. Walter P. (2009). Membrane expansion alleviates endoplasmic reticulum stress independently of the unfolded protein response. *J. Cell Biol.* 187 525–536. 10.1083/jcb.200907074 19948500 PMC2779237

[B71] Sezgin E. Levental I. Mayor S. Eggeling C. (2017). The mystery of membrane organization: composition, regulation and roles of lipid rafts. *Nat. Rev. Mol. Cell Biol.* 18 361–374. 10.1038/nrm.2017.16 28356571 PMC5500228

[B72] Sherman F. (2002). Getting started with yeast. *Methods Enzymol.* 350 3–41. 10.1016/S0076-6879(02)50954-X 12073320

[B73] Shevchenko A. Simons K. (2010). Lipidomics: coming to grips with lipid diversity. *Nat. Rev. Mol. Cell Biol.* 11 593–598. 10.1038/nrm2934 20606693

[B74] Stordeur C. Puth K. Sáenz J. P. Ernst R. (2014). Crosstalk of lipid and protein homeostasis to maintain membrane function. *Biol. Chem.* 395 313–326. 10.1515/hsz-2013-0235 24152902

[B75] Stukey J. E. McDonough V. M. Martin C. E. (1989). Isolation and characterization of OLE1, a gene affecting fatty acid desaturation from *Saccharomyces cerevisiae*. *J. Biol. Chem.* 264 16537–16544. 2674136

[B76] Sumper M. Riepertinger C. Lynen F. Oesterhelt D. (1969). Die Synthese verschiedener Carbonsäuren durch den Multienzymkomplex der Fettsäuresynthese aus Hefe und die Erklärung ihrer Bildung. *Eur. J. Biochem.* 10 377–387. 10.1111/j.1432-1033.1969.tb00701.x 4390422

[B77] Surma M. A. Herzog R. Vasilj A. Klose C. Christinat N. Morin-Rivron D. (2015). An automated shotgun lipidomics platform for high throughput, comprehensive, and quantitative analysis of blood plasma intact lipids. *Eur. J. Lipid Sci. Technol* 117 1540–1549. 10.1002/ejlt.201500145 26494980 PMC4606567

[B78] Surma M. A. Klose C. Klemm R. W. Ejsing C. S. Simons K. (2011). Generic sorting of raft lipids into secretory vesicles in yeast. *Traffic* 12 1139–1147. 10.1111/j.1600-0854.2011.01221.x 21575114

[B79] Surma M. A. Klose C. Peng D. Shales M. Mrejen C. Stefanko A. (2013). A lipid E-MAP identifies Ubx2 as a critical regulator of lipid saturation and lipid bilayer stress. *Mol. Cell* 51 519–530. 10.1016/j.molcel.2013.06.014 23891562 PMC3791312

[B80] Tabas I. Ron D. (2011). Integrating the mechanisms of apoptosis induced by endoplasmic reticulum stress. *Nat. Cell Biol.* 13 184–190. 10.1038/ncb0311-184 21364565 PMC3107571

[B81] Thibault G. Shui G. Kim W. McAlister G. C. Ismail N. Gygi S. P. (2012). The membrane stress response buffers lethal effects of lipid disequilibrium by reprogramming the protein homeostasis network. *Mol. Cell* 48 16–27. 10.1016/j.molcel.2012.08.016 23000174 PMC3496426

[B82] Tran D. M. Ishiwata-Kimata Y. Mai T. C. Kubo M. Kimata Y. (2019). The unfolded protein response alongside the diauxic shift of yeast cells and its involvement in mitochondria enlargement. *Sci. Rep.* 9:12780. 10.1038/s41598-019-49146-5 31484935 PMC6726593

[B83] Travers K. J. Patil C. K. Wodicka L. Lockhart D. J. Weissman J. S. Walter P. (2000). Functional and genomic analyses reveal an essential coordination between the unfolded protein response and ER-associated degradation. *Cell* 101 249–258. 10.1016/S0092-8674(00)80835-1 10847680

[B84] Uhlén M. Fagerberg L. Hallström B. M. Lindskog C. Oksvold P. Mardinoglu A. (2015). Tissue-based map of the human proteome. *Science* 347:1260419. 10.1126/science.1260419 25613900

[B85] van Meer G. Voelker D. R. Feigenson G. W. (2008). Membrane lipids: where they are and how they behave. *Nat. Rev. Mol. Cell Biol.* 9 112–124. 10.1038/nrm2330 18216768 PMC2642958

[B86] Volmer R. Ron D. (2015). Lipid-dependent regulation of the unfolded protein response. *Curr. Opin. Cell Biol.* 33 67–73. 10.1016/j.ceb.2014.12.002 25543896 PMC4376399

[B87] Walter P. Ron D. (2011). The unfolded protein response: from stress pathway to homeostatic regulation. *Science* 334 1081–1086. 10.1126/science.1209038 22116877

[B88] Wang M. Kaufman R. J. (2014). The impact of the endoplasmic reticulum protein-folding environment on cancer development. *Nat. Rev. Cancer* 14 581–597. 10.1038/nrc3800 25145482

[B89] Wang S. Kaufman R. J. (2012). The impact of the unfolded protein response on human disease. *J. Cell Biol.* 197 857–867. 10.1083/jcb.201110131 22733998 PMC3384412

[B90] Wolf D. H. Schäfer A. (2006). CPY* and the power of yeast genetics in the elucidation of quality control and associated protein degradation of the endoplasmic reticulum. *Curr. Top. Microbiol. Immunol.* 300 41–56. 10.1007/3-540-28007-3_3 16573236

[B91] Xu H. Huang C. H. (1987). Scanning calorimetric study of fully hydrated asymmetric phosphatidylcholines with one acyl chain twice as long as the other. *Biochemistry* 26 1036–1043. 10.1021/bi00378a009 3567154

