# Supplementary material for: A Quantitative Analysis of Cellular Lipid Compositions During Acute Proteotoxic ER Stress Reveals Specificity in the Production of Asymmetric Lipids
Source: Front Cell Dev Biol. 2020 Aug 4;8:756. doi: 10.3389/fcell.2020.00756 (PMC7417482; doi:10.3389/fcell.2020.00756)
Supplement: Supplementary file 1 [file Data_Sheet_1.PDF]

Supplementary Material

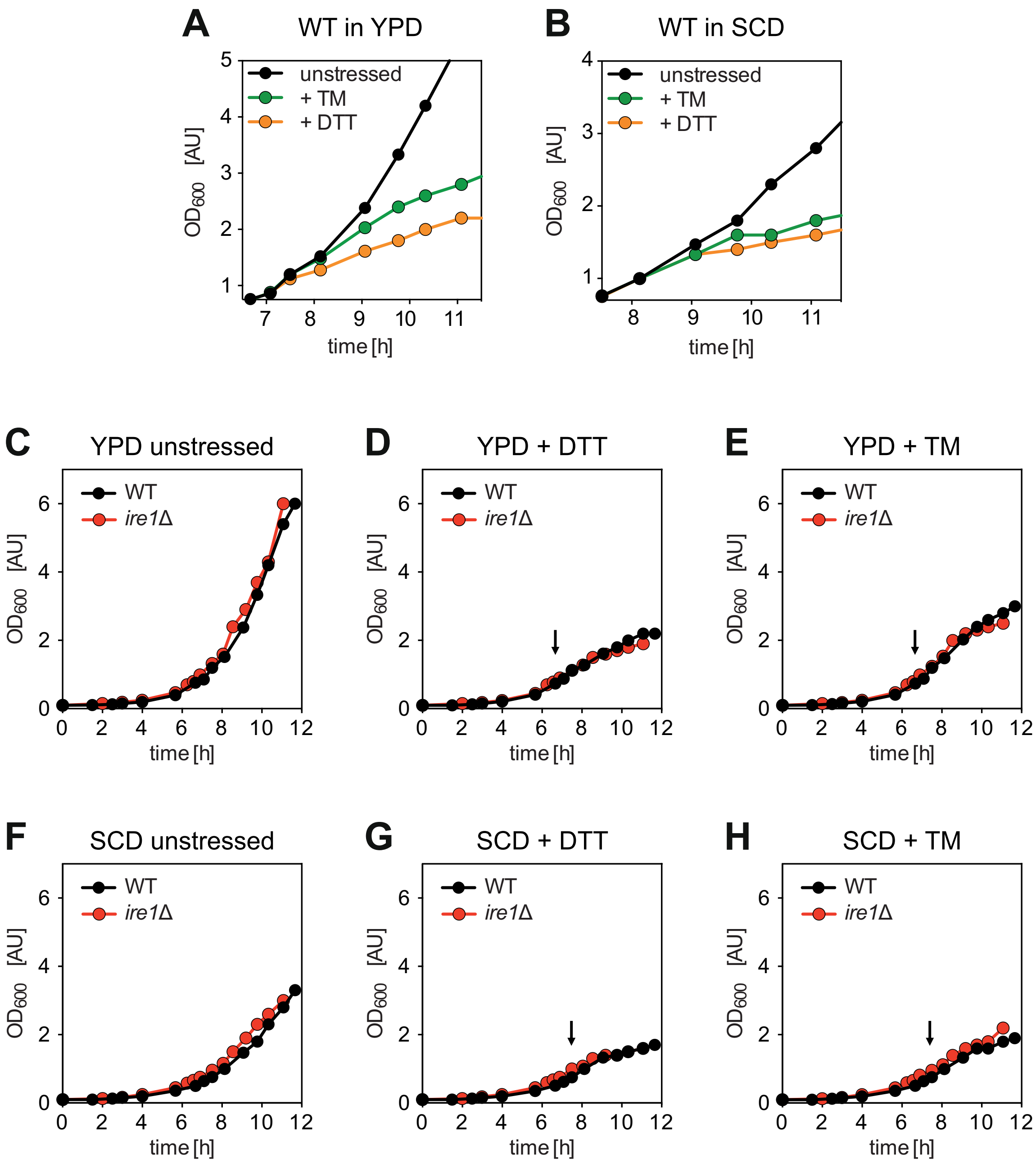

Supplementary Figure S1. Acute growth defect by UPR inducing drugs is predominantly a consequence of proteotoxic stress independent of UPR activation. (A),(B) The data are replotted from Figure 1E-F. Only a subset of data is represented with a particular focus on the period the cells were either stressed by DTT (orange), TM (green) or left untreated (black). The first datapoint corresponds to the moment of adding the proteotoxic drug. **(C)-(H)** The data are replotted from Figure 1E-H to highlight the similar growth of BY4741 WT and *ire1*Δ cells. The arrow indicates the moment of adding the proteotoxic drug (6.7 h and 7.5 h in YPD and SCD, respectively). All data in in this Figure are derived from a single, representative experiment.

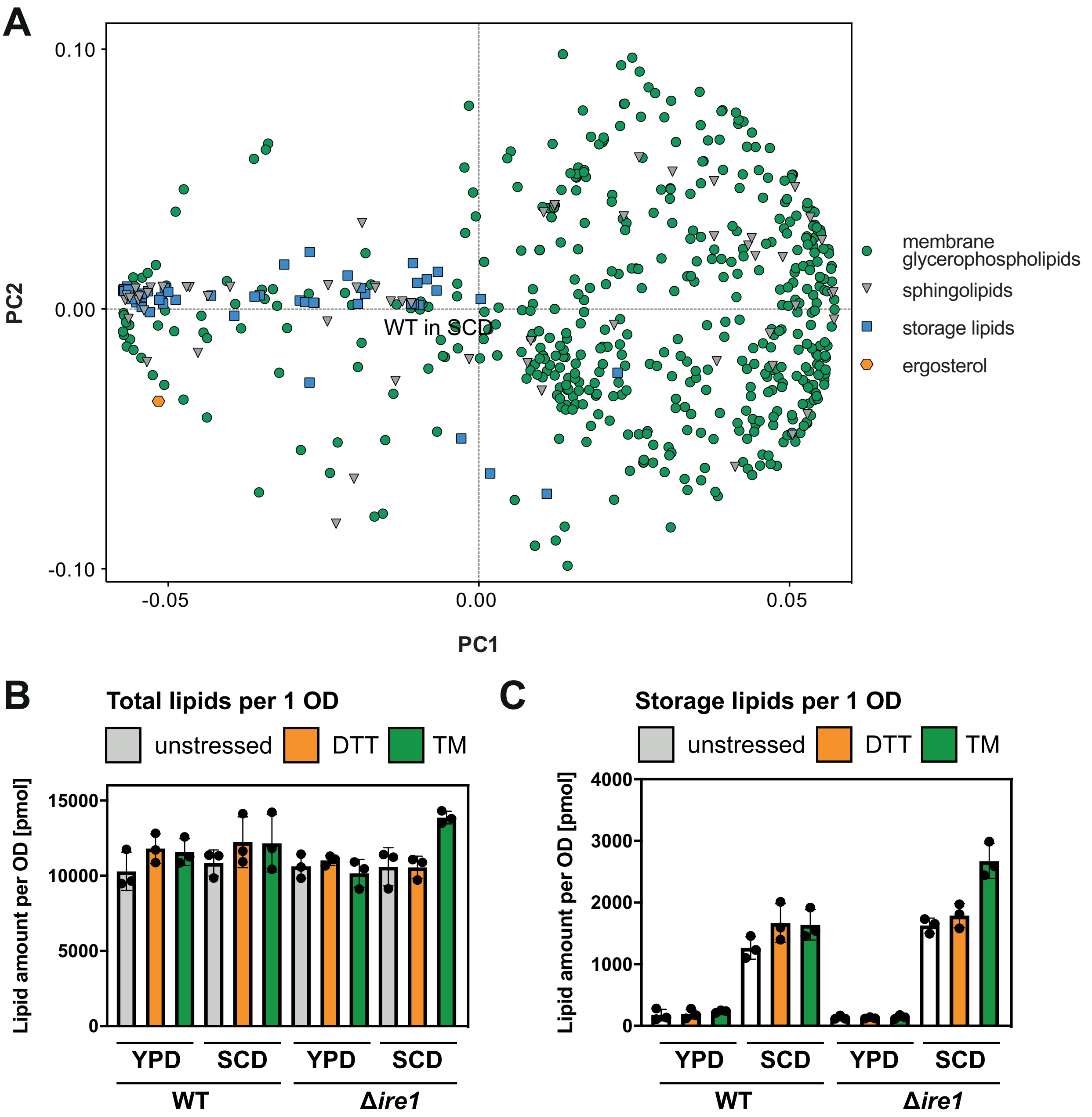

Supplementary Figure S2. Loadings plot of the entire dataset. (**A**) The loadings plots indicate the contribution (loadings) of individual lipid species to the principle components 1 and 2 from Figure 2B. For clarity, only membrane glycerolipids with two acyl chains were considered. The data suggest low, negative loadings of most storage lipids (SL, blue squares), sphingolipids (gray triangles) and ergosterol (orange hexagons) on principle component 1. In contrast, the majority of membrane glycerophospholipids (green circles) have low, positive loadings on principle component 1. (**B**),(**C**) The total amount of lipids (A) and the amount of storage lipids (B) quantified from 1 OD unit of cells is plotted for different cells and media as indicated. The color code indicates unstressed (gray), DTT-stressed (orange), and TM-stressed (green) cells.

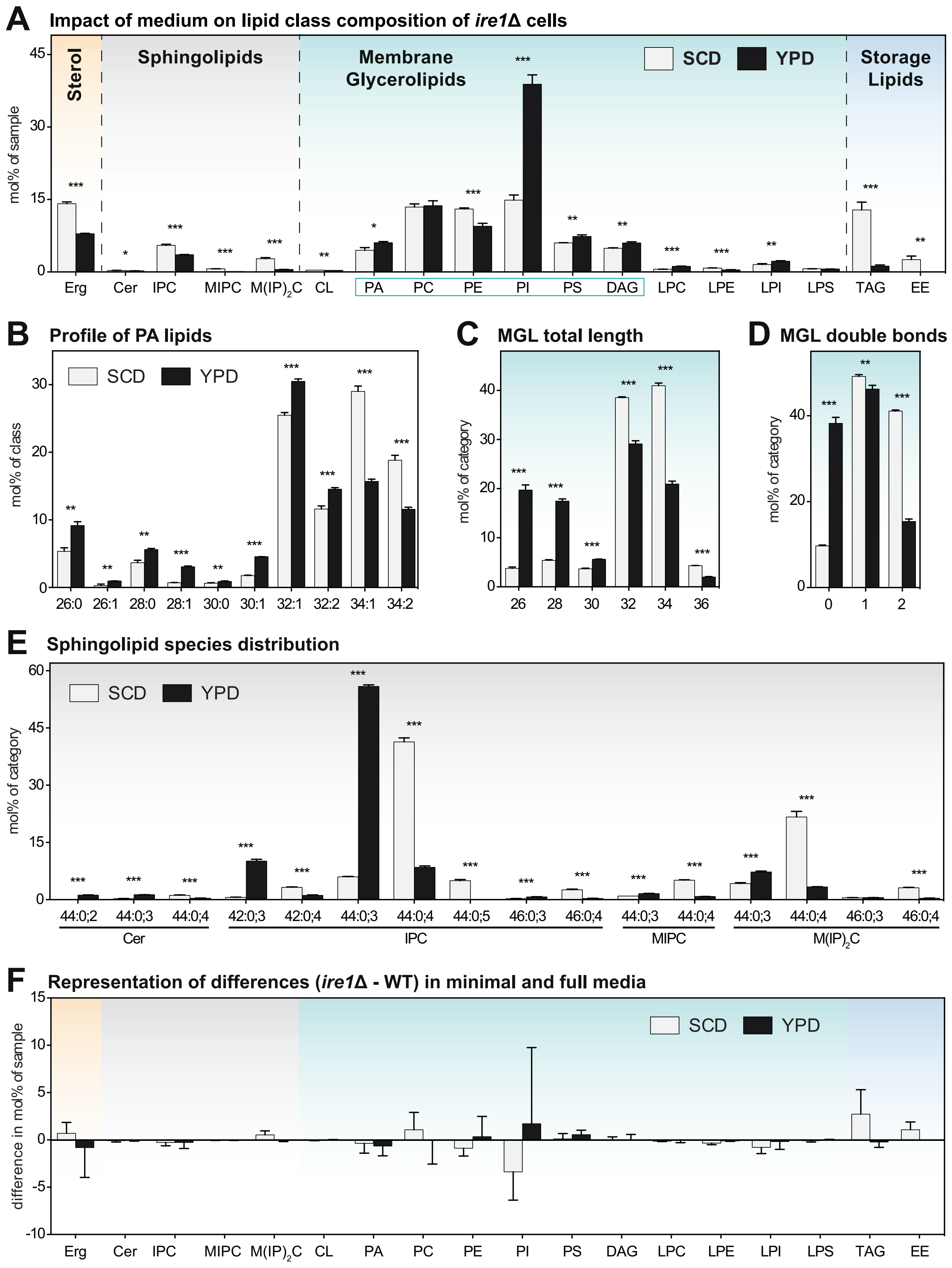
A
Impact of medium on lipid class composition of ire1Δ cells
Sterol
Sphingolipids
Membrane Glycerolipids
Storage Lipids
SCD
YPD
mol% of sample
Erg
Cer
IPC
MIPC
M(IP)2C
CL
PA
PC
PE
PI
PS
DAG
LPC
LPE
LPI
LPS
TAG
EE
B
Profile of PA lipids
mol% of class
26:0
26:1
28:0
28:1
30:0
30:1
32:1
32:2
34:1
34:2
C
MGL total length
mol% of category
D
MGL double bonds
E
Sphingolipid species distribution
Cer
IPC
MIPC
M(IP)2C
F
Representation of differences (ire1Δ - WT) in minimal and full media
difference in mol% of sample

Supplementary Figure S3. The lipid composition of *ire1*Δ cells in different media. A single colony of *ire1*Δ cells was used to inoculate a preculture in either minimal (SCD) or full (YPD) medium. After overnight cultivation for 21 hours, a fresh culture was inoculated to an OD_{600} of 0.1. When the cells reached an OD_{600} of 0.80 ± 0.05, they were cultivated for one more hour. 20 OD equivalents of these cells were harvested and analyzed by lipid mass spectrometry. The data represented by black and white bars relate to cells cultivated in full and minimal medium, respectively. **(A)** Lipid class composition in mol% of all quantified lipids in the sample organized by lipid categories. **(B)** Profile of PA lipids in mol% of the class. **(C)** Total length of lipids in a sub-category of MGLs (PA, PC, PE, PI, PS, DAG). The total length is given as the sum of carbon atoms in both fatty acyl chains in mol% of this sub-category. **(D)** Total number double bonds in a sub-category of MGLs (PA, PC, PE, PI, PS, DAG) is given as the sum of double bonds in both acyl chains and represented in mol% of this sub-category. **(E)** Profile of sphingolipids in mol% of this category. **(F)** The difference in lipid class abundance in mol% between WT and *ire1*Δ is both SCD and YPD was determined and is plotted. The difference was calculated by subtracting the lipid class abundance in WT cells from the abundance in *ire1*Δ under consideration of error propagation. The least abundant species in each panel are omitted for clarity. Each bar represents the average ± SD from n = 3 independent experiments. Statistical significance was tested by an unpaired two-tailed t-test using GraphPad Prism, $^{*}p<0.05$, $^{**}p<0.01$, $^{***}p<0.001$.

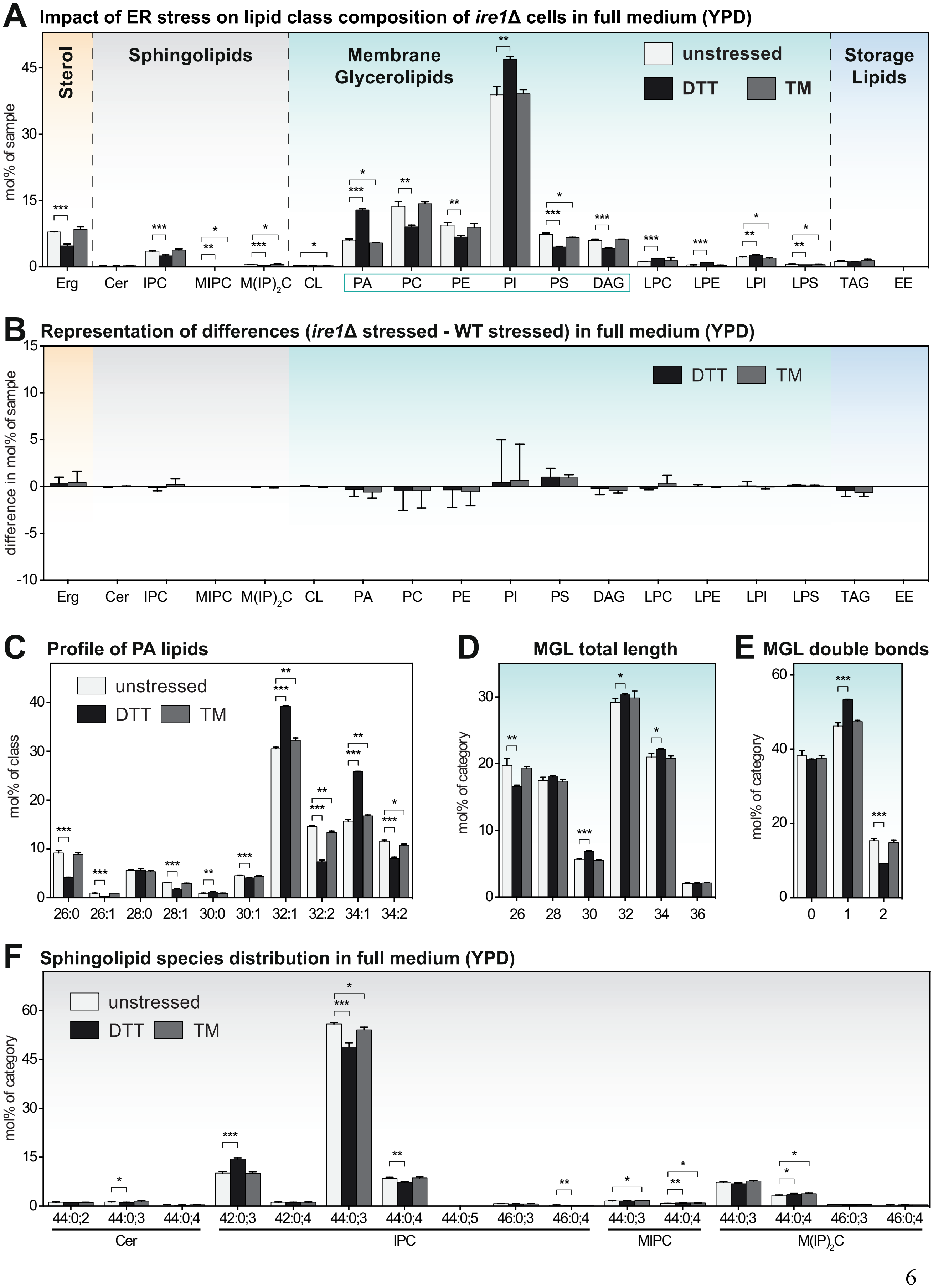
A Impact of ER stress on lipid class composition of ire1Δ cells in full medium (YPD)
Sterol
Sphingolipids
Membrane Glycerolipids
Storage Lipids
unstressed
DTT
TM
mol% of sample
Erg
Cer
IPC
MIPC
M(IP)2C
CL
PA
PC
PE
PI
PS
DAG
LPC
LPE
LPI
LPS
TAG
EE
B Representation of differences (ire1Δ stressed - WT stressed) in full medium (YPD)
difference in mol% of sample
C Profile of PA lipids
mol% of class
26:0
26:1
28:0
28:1
30:0
30:1
32:1
32:2
34:1
34:2
D MGL total length
mol% of category
E MGL double bonds
F Sphingolipid species distribution in full medium (YPD)
44:0;2
44:0;3
44:0;4
42:0;3
42:0;4
44:0;5
46:0;3
46:0;4
Cer
IPC
MIPC
M(IP)2C

Supplementary Figure S4. The impact of proteotoxic stress on the lipidome of *ire1*Δ cells in full medium. A single colony of *ire1*Δ cells was used to inoculate a preculture in full (YPD) medium. After overnight cultivation for 21 hours, a fresh culture was inoculated to an OD_{600} of 0.1 and then cultivated to an OD_{600} of 0.80 ± 0.05. The cells were then either left untreated (white bars), stressed by the addition of either with 8 mM DTT (black bars) or 1.0 µg/ml TM (gray bars). After one additional hour of cultivation, 20 OD equivalents of these cells were harvested and analyzed by lipid mass spectrometry. **(A)** Lipid class composition in mol% of all quantified lipids in the sample organized by lipid categories. **(B)** The difference in lipid class abundance in stressed minus unstressed cells highlights the impact of DTT (black) and TM (gray) on the cellular lipid composition in full medium. The difference in abundance in mol% was calculated by subtracting the abundance in unstressed cells from the abundance in either DTT- or TM-stressed cells under consideration of error propagation. **(C)** Profile of PA lipids in mol% of the class. **(D)** Total length of lipids in a sub-category of MGLs (PA, PC, PE, PI, PS, DAG). The total length is given as the sum of carbon atoms in both fatty acyl chains in mol% of this sub-category. **(E)** Total number double bonds in a sub-category of MGLs (PA, PC, PE, PI, PS, DAG) is given as the sum of double bonds in both acyl chains and represented in mol% of this sub-category. **(F)** Profile of sphingolipids in mol% of this category. The least abundant species are omitted for clarity. Each bar represents the average ± SD from n = 3 independent experiments. Statistical significance was tested by an unpaired two-tailed t-test using GraphPad Prism, *$p<0.05$, **$p<0.01$, ***$p<0.001$.

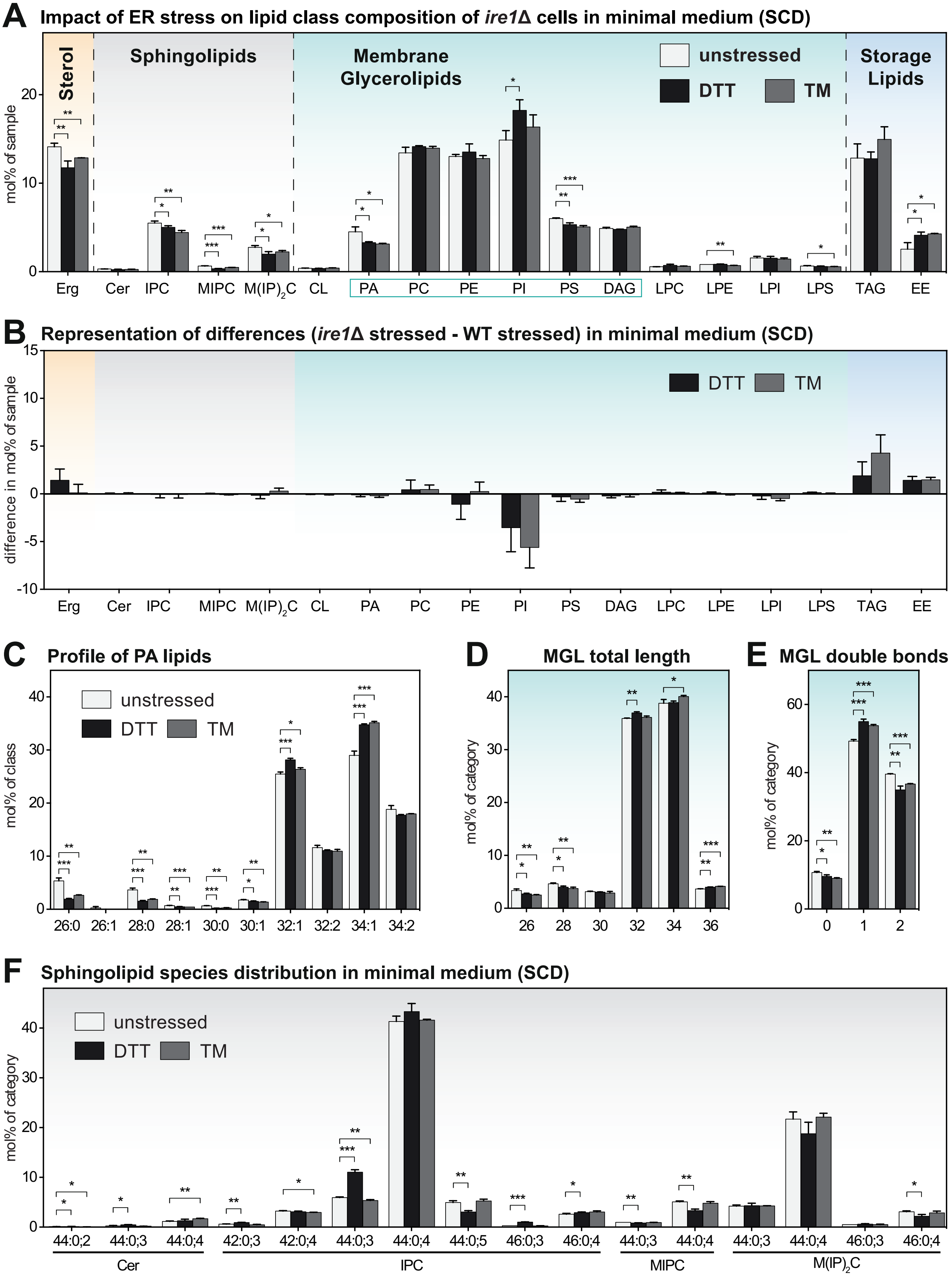
A Impact of ER stress on lipid class composition of ire1Δ cells in minimal medium (SCD)
Sterol
Sphingolipids
Membrane Glycerolipids
Storage Lipids
unstressed
DTT
TM
mol% of sample
Erg Cer IPC MIPC M(IP)2C CL PA PC PE PI PS DAG LPC LPE LPI LPS TAG EE
B Representation of differences (ire1Δ stressed - WT stressed) in minimal medium (SCD)
difference in mol% of sample
DTT
TM
Erg Cer IPC MIPC M(IP)2C CL PA PC PE PI PS DAG LPC LPE LPI LPS TAG EE
C Profile of PA lipids
mol% of class
26:0 26:1 28:0 28:1 30:0 30:1 32:1 32:2 34:1 34:2
D MGL total length
mol% of category
26 28 30 32 34 36
E MGL double bonds
mol% of category
0 1 2
F Sphingolipid species distribution in minimal medium (SCD)
mol% of category
44:0;2 44:0;3 44:0;4 42:0;3 42:0;4 44:0;3 44:0;4 44:0;5 46:0;3 46:0;4 44:0;3 44:0;4 44:0;3 44:0;4 46:0;3 46:0;4
Cer
IPC
MIPC
M(IP)2C

Supplementary Figure S5 The impact of proteotoxic stress on the lipidome of *ire1*Δ cells in minimal medium. A single colony of *ire1*Δ cells was used to inoculate a preculture in minimal (SCD) medium. After overnight cultivation for 21 hours, a fresh culture was inoculated to an OD_{600} of 0.1 and then cultivated to an OD_{600} of 0.80 ± 0.05. The cells were then either left untreated (white bars), stressed by the addition of either with 8 mM DTT (black bars) or 1.0 µg/ml TM (gray bars). After one additional hour of cultivation, 20 OD equivalents of these cells were harvested and analyzed by lipid mass spectrometry. **(A)** Lipid class composition in mol% of all quantified lipids in the sample organized by lipid categories. **(B)** The difference in lipid class abundance in stressed minus unstressed cells highlights the impact of DTT (black) and TM (gray) on the cellular lipid composition in minimal medium. The difference in abundance in mol% was calculated by subtracting the abundance in unstressed cells from the abundance in either DTT- or TM-stressed cells under consideration of error propagation. **(C)** Profile of PA lipids in mol% of the class. **(D)** Total length of lipids in a sub-category of MGLs (PA, PC, PE, PI, PS, DAG). The total length is given as the sum of carbon atoms in both fatty acyl chains in mol% of this sub-category. **(E)** Total number double bonds in a sub-category of MGLs (PA, PC, PE, PI, PS, DAG) is given as the sum of double bonds in both acyl chains and represented in mol% of this sub-category. **(F)** Profile of sphingolipids in mol% of this category. The least abundant species are omitted for clarity. Each bar represents the average ± SD from n = 3 independent experiments. Statistical significance was tested by an unpaired two-tailed t-test using GraphPad Prism, *$p<0.05$, **$p<0.01$, ***$p<0.001$.
